# Is environmental behavior related to economic risk preferences? An exploratory case by case analysis

**DOI:** 10.3389/fpsyg.2023.1212685

**Published:** 2023-08-03

**Authors:** Stepan Vesely, Christian A. Klöckner

**Affiliations:** Institute of Psychology, Norwegian University of Science and Technology, Trondheim, Norway

**Keywords:** environmental behavior, consumer behavior, risk preferences, attitude towards risk, economic preferences

## Abstract

Do risk preferences play a role when deciding whether to act pro-environmentally? Looking at 28 different behaviors case by case – including recycling, waste reduction, energy and water conservation, consumer behavior, and environmental policy support – our data suggest no relation between most of the behaviors and economic risk preferences. However, economic risk preferences appear to have some relevance for travel mode choice and for specific consumer preferences (eco-friendly detergents, organic food, and single-use plastics), perhaps because people are better able to appreciate aspects of these behaviors related to risk (e.g., possibility of traffic accidents, health risks).

## 1. Introduction and research background

A number of recent papers investigate whether “social” and “economic” preferences, such as altruism, reciprocity, and risk and time preferences may be related to people’s environmental behavior (Schleich et al., 2019; Fischbacher et al., 2021; Lades et al., 2021; Ziegler, 2021; Andre et al., 2022).

Risk preferences are a widely studied topic in psychology and economics, and can be viewed as a person’s tendency to choose more or less risky outcomes (termed risk seeking and risk aversion, respectively). In this paper, we assess “economic” risk preferences by letting participants choose between small financial gambles involving different degrees of risk. The advantage of this approach is its simplicity, ease of administration, and mitigation of hypothetical bias (as participants are paid according to their choices). While measuring risk preferences in this way is common (e.g., Holt and Laury, 2002), the approach only captures one aspect of how people decide under risk. Decision-making under risk is generally more complex (e.g., Slovic, 1964; Frey et al., 2023). It is therefore useful to point out at the outset that our study only looks at one slice of the potentially more complex relationship between environmental behavior and risk preferences more generally (including, but not limited to economic risk preferences). Several models of environmental behavior have been developed and tested in recent decades (Klöckner, 2013). Although some environmental psychology models incorporate the distinct but complementary concept of risk perception (van der Linden, 2015), no major model of environmental behavior currently incorporates risk preferences as an individual difference variable. This can lead to biased estimates of the influence of the factors that are modeled due to omitted variable bias (Ziegler, 2021). To avoid omitted variable bias, risk preferences may therefore need to be considered when modeling environmental behavior.

Indeed, prior research suggests that risk preferences are related to some environmental behaviors, namely energy efficiency investments (Qiu et al., 2014; Volland, 2017; He et al., 2019; Olsthoorn et al., 2019; Schleich et al., 2019; Fischbacher et al., 2021; Kim and Nam, 2021; but see Lades et al., 2021), donations to environmental organizations (Ziegler, 2021; but see Andre et al., 2022), organic food consumption (Li et al., 2021), and avoiding plastic waste (Fuhrmann-Riebel et al., 2021). Risk preferences appear not to be robustly related to energy consumption (Fischbacher et al., 2021; Fuhrmann-Riebel et al., 2021; Groh and Ziegler, 2022; however, see Volland, 2017), preferences for green electricity (Ziegler, 2020; but see Petrovich et al., 2021), or to broad indices of environmental behavior (Naderi and Van Steenburg, 2018; Lades et al., 2021). A key limitation of existing research on the role of risk preferences in the environmental domain is the narrow scope of most previous studies focusing only on a few examples of pro-environmental conduct at a time (for an exception see Lades et al., 2021). This means that areas of environmental behavior shaped by a person’s risk preferences can be easy to miss. To generate more systematic evidence, the present study therefore tests if risk preferences are related to any of a set of 28 different environmental behaviors. To preview our results, we indeed find instances of environmental behavior correlated with risk preferences that have been overlooked in previous research.

Because of the wide variety of environmental behaviors, it is unclear whether studying just a few selected behavior examples can lead to generalizable findings concerning the role played by risk preferences (it is of course also possible that the influence of risk preferences does not generalize to most or even many environmental behaviors, which in fact is what our results suggest). A related issue is that reliance on general behavior indices or psychometric scales constructed by pooling participants’ responses across many environmental behaviors may similarly not be optimal when investigating the role of risk preferences, as correlations between general behavior scales and economic and social preferences may conceal important links between specific behaviors and specific preferences (see Lades et al., 2021). Taking a case by case approach that examines whether risk preferences correlate with a broad selection of specific behaviors is therefore warranted. This approach will allow us to more systematically evaluate which environmental behaviors might be affected by risk preferences, highlighting behavioral domains which may be subsequently prioritized in confirmatory research.

## 2. Methods

We report exploratory analyses based on data collected as part of a larger study reported in Vesely et al. (2020).

### 2.1. Participants and statistical power

Two hundred and eight participants (111 women; mean age = 23.9 years, SD = 5.5 years) recruited from a subject pool maintained by the Vienna Center for Experimental Economics took part in the study, programmed in z-Tree (Fischbacher, 2007). Participants were compensated for their time, earning 35.3 EUR on average. *A priori* power calculation based on Faul et al. (2007) indicated that a sample of at least 191 participants was required to detect small linear associations – partial *R*^2^ = 0.04, with alpha at 0.05 (two-sided) and statistical power at 0.80.

### 2.2. Measures

Measures relevant for the present study are described below. Printscreens of the complete instructions are provided in online Appendix A.

#### 2.2.1. Risk preferences

We elicited participants’ risk preferences using a task adapted from Eckel and Grossman (2002), in which participants were asked to choose one of eight incentivized lotteries involving various degrees of risk. An example of a low risk lottery was a prospect of getting either 4.2 EUR or 6.0 EUR with equal probability, while an example of a high risk lottery was a prospect of getting either 1.4 EUR or 10.8 EUR with equal probability. Participants were assigned a risk seeking score which was equal to 1 if the safest lottery was selected and equal to 8 if the most risky lottery was selected, with values in between for lotteries involving intermediate levels of risk. Therefore, higher values on the Eckel and Grossman measure indicate more risk seeking preferences. Conversely, lower values indicate more risk averse preferences. Page 14 in online Appendix A shows the risk elicitation decision screen.

#### 2.2.2. Environmental behaviors

Participants responded to 28 questions about their previous pro-environmental behaviors. Most of the items were adapted from Kaiser (1998). See online Appendix B for the full set of items and the coding of response options. Participants’ responses are scored so that higher scores indicate performing pro-environmental behavior.

## 3. Results

We report rank-biserial correlations (with jackknife standard errors) between each environmental behavior and participants’ risk preferences in Table 1. As a result of how our behavior and risk preference measures are scored, positive correlations between a behavior and the risk preference measure indicate that risk seekers are more likely to perform the behavior.

**Table 1:** Correlations between environmental behaviors and risk preferences.

Environmental behavior label	*r*_rb_ (std. err.)
Public transportation	0.33***^a^ (0.07)
Organic dairy	0.16^†^ (0.09)
Organic meat	0.15^†^ (0.08)
Voting	0.14^†^ (0.09)
Green electricity	0.14 (0.14)
Taxes	0.07 (0.10)
Showering	0.07 (0.14)
Refrigerator	0.06 (0.10)
Recycling paper	0.05 (0.10)
Laundry	0.05 (0.13)
Fuel efficiency	0.02 (0.08)
Eating meat	0.02 (0.10)
Room temperature	0.02 (0.14)
Friends	0.00 (0.08)
Composting	−0.01 (0.08)
Driving defensively	−0.01 (0.11)
Environmental organization	−0.01 (0.14)
Dryer	−0.05 (0.09)
Heater	−0.06 (0.08)
Reusable bottles	−0.06 (0.08)
Local food	−0.09 (0.11)
Electronic devices	−0.11 (0.08)
Incentives	−0.11 (0.08)
Standby	−0.11 (0.08)
Batteries	−0.18^†^ (0.10)
Reusable cups	−0.22* (0.10)
Detergent	−0.28**^a^ (0.09)
Shopping bags	−0.63^†^ (0.38)

^†^ < 0.1, *p < 0.05, **p < 0.01, ***p < 0.001 (all tests are two-sided).

^a^This being exploratory research, *p*-values are not corrected for multiple hypothesis testing. However, estimates marked with an “a” superscript would still be significant were we to apply the Benjamini and Hochberg (1995) procedure.

Results show that risk preferences are virtually unrelated or only weakly related to most environmental behaviors, including recycling (items Recycling paper, Composting), water conservation (items Showering, Laundry), energy conservation (items Laundry, Heater, Standby, Room temperature, Electronic devices, Refrigerator, Dryer), preferences for green electricity and for low-emission cars (items Green electricity, Fuel efficiency), and support for fiscal policy measures (items Taxes, Incentives).

In contrast, there is evidence that risk seekers more commonly use alternative travel modes like walking, cycling, and public transportation (item Public transportation). Risk seekers are, however, also more likely to use eco-unfriendly detergents (item Detergent) and more likely to use single-use cups (item Reusable cups). The three correlations are moderate in size.

There is also tentative evidence that risk seekers are more likely to eat organic food (items Organic dairy, Organic meat) and more likely to vote for political parties with environmental topics high on their agenda (item Voting). On the other hand, risk averse people are more likely to return dead batteries to collection points for dangerous waste (item Batteries) and to bring their own bags when shopping (item Shopping bags). These correlations are not statistically significant at conventional levels and are generally small, except for the correlation with Shopping bags, which is large but imprecisely estimated.

## 4. Concluding remarks

The picture that emerges from our exploratory analyses suggests a limited importance of risk preferences in the environmental behavior domain as a whole, a conclusion that is broadly in line with Lades et al. (2021). We nevertheless highlight specific environmental behaviors where more research on the possible role of risk preferences is warranted and tentatively suggest the following new hypotheses:

Risk aversion may lead people to behave more pro-environmentally when the target behavior mitigates personal or public health risks (e.g., using eco-friendly detergents or getting rid of e-waste properly). However, this pattern is not apparent when it comes to food consumption, as risk averse people may be less likely to eat organic.

Additionally, risk aversion may discourage pro-environmental behavior when this is associated with exposure to outside conditions (e.g., using public transportation or cycling to work). Risk aversion may, on the other hand, motivate pro-environmental behavior when this in some way limits exposure to outside conditions (e.g., bringing own shopping bags or reusable cups).

This study illustrates the usefulness of assessing the relationship between environmental behavior and risk preferences on a case by case basis. Nevertheless, a number of limitations and possible extensions can be noted.

Environmental behavior issues. While we examined possible links of several different environmental behaviors with risk preferences, subsequent research may approach this question even more systematically and include additional environmental behaviors beyond those covered here (Lange and Dewitte, 2019). Subsequent research may similarly investigate the possible existence of clusters of related behaviors all linked to risk preferences (Lades et al., 2021). We relied on a dichotomous response format when assessing environmental behavior, which fails to capture variance in the intensity or frequency of engagement in specific behaviors and follow-up studies may benefit from using response scales better suited for that purpose.

Risk preferences issues. We recommend that the operationalization of risk preferences via choice among risky financial prospects be complemented by alternative operationalizations capturing other important aspects of risk preferences (Blais and Weber, 2006). In addition, to assess the unique role of risk preferences in environmental decision making, it would be desirable to account for the influence of other types of economic preferences (Schleich et al., 2019; Lades et al., 2021). Further, assessments of risk perceptions (Wilson et al., 2019) and risk preferences may be profitably combined when modeling environmental choices; one can envision that the two factors may interact, with risk preferences coming to the fore particularly when the behavior at hand is in fact perceived to involve risky outcomes.

Since risk preferences were measured after environmental behavior in this study, we also cannot rule out the possibility of order effects, although we do not believe them to be especially likely. Finally, subsequent confirmatory studies should recruit relatively large samples, as the present study had barely enough power to distinguish the modest correlations with risk preferences from noise.

In summary, our approach of looking at the different behaviors case by case allowed us to start systematically uncovering the role that risk preferences sometimes play when deciding whether to behave pro-environmentally. We interpret our results cautiously, viewing them as a starting point for theory building and confirmatory research. If our findings are confirmed, we hope this will lead to incorporating risk preferences into environmental behavior models when appropriate – for example in the area of travel mode choice. However, this exploratory study also provides initial evidence that in most instances incorporating economic risk preferences into environmental behavior models may not be necessary.

## Data availability statement

The raw data supporting the conclusions of this article will be made available by the authors, without undue reservation.

## Ethics statement

Ethical review and approval was not required for the study on human participants in accordance with the local legislation and institutional requirements. The participants provided their written informed consent to participate in this study.

## Author contributions

All authors listed have made a substantial, direct, and intellectual contribution to the work and approved it for publication.

## Conflict of interest

The authors declare that the research was conducted in the absence of any commercial or financial relationships that could be construed as a potential conflict of interest.

## Publisher’s note

All claims expressed in this article are solely those of the authors and do not necessarily represent those of their affiliated organizations, or those of the publisher, the editors and the reviewers. Any product that may be evaluated in this article, or claim that may be made by its manufacturer, is not guaranteed or endorsed by the publisher.
