# Supplementary figures and images for: Is environmental behavior related to economic risk preferences? An exploratory case by case analysis
Source: Front Psychol. 2023 Aug 3;14:1212685. doi: 10.3389/fpsyg.2023.1212685 (PMC10434762; doi:10.3389/fpsyg.2023.1212685)

Online Appendix A: Printscreens of instructions
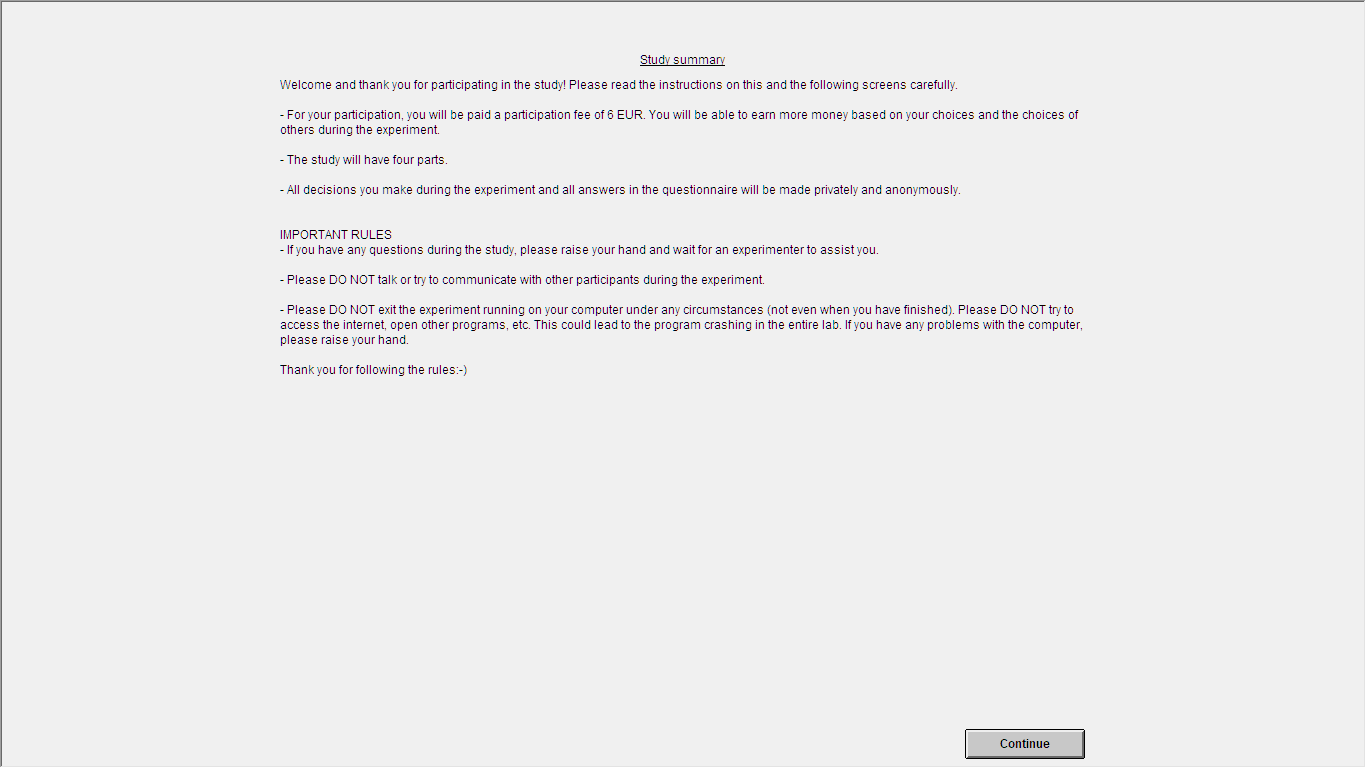

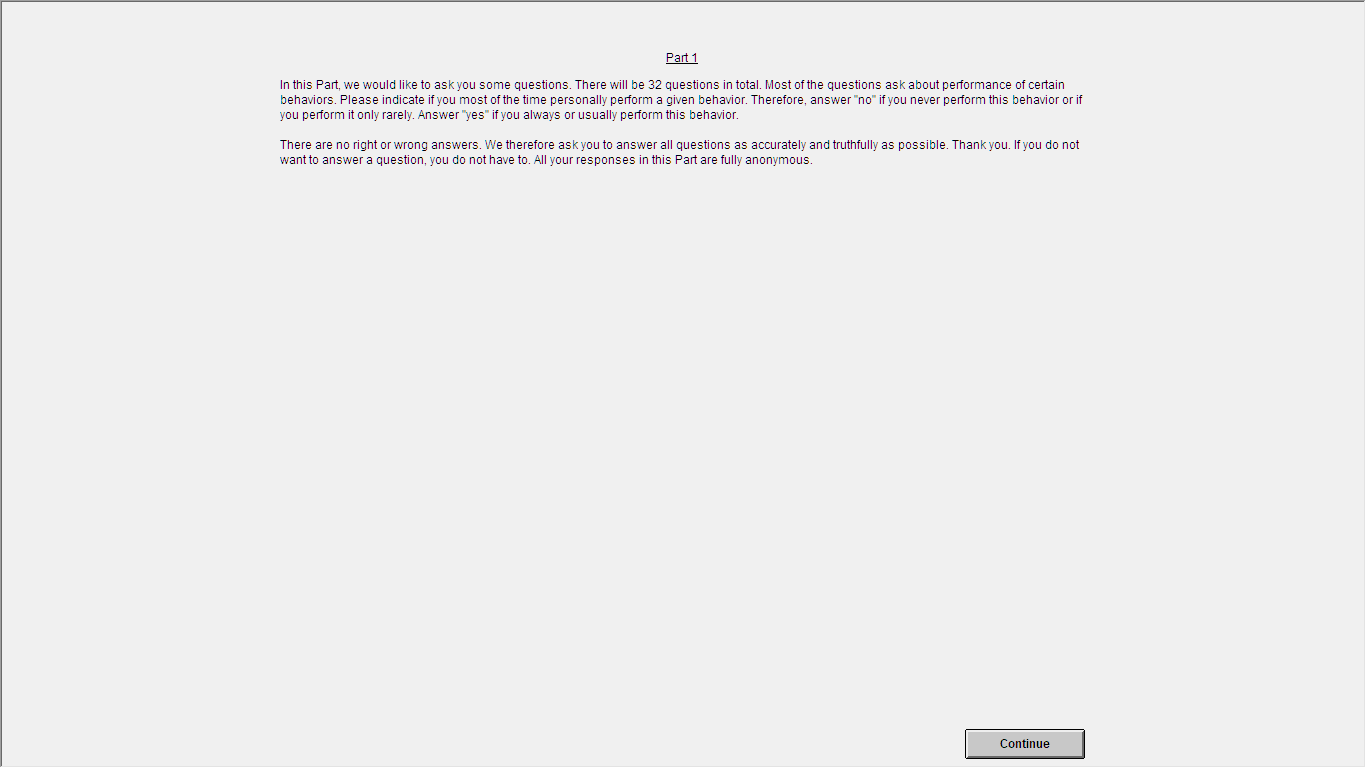

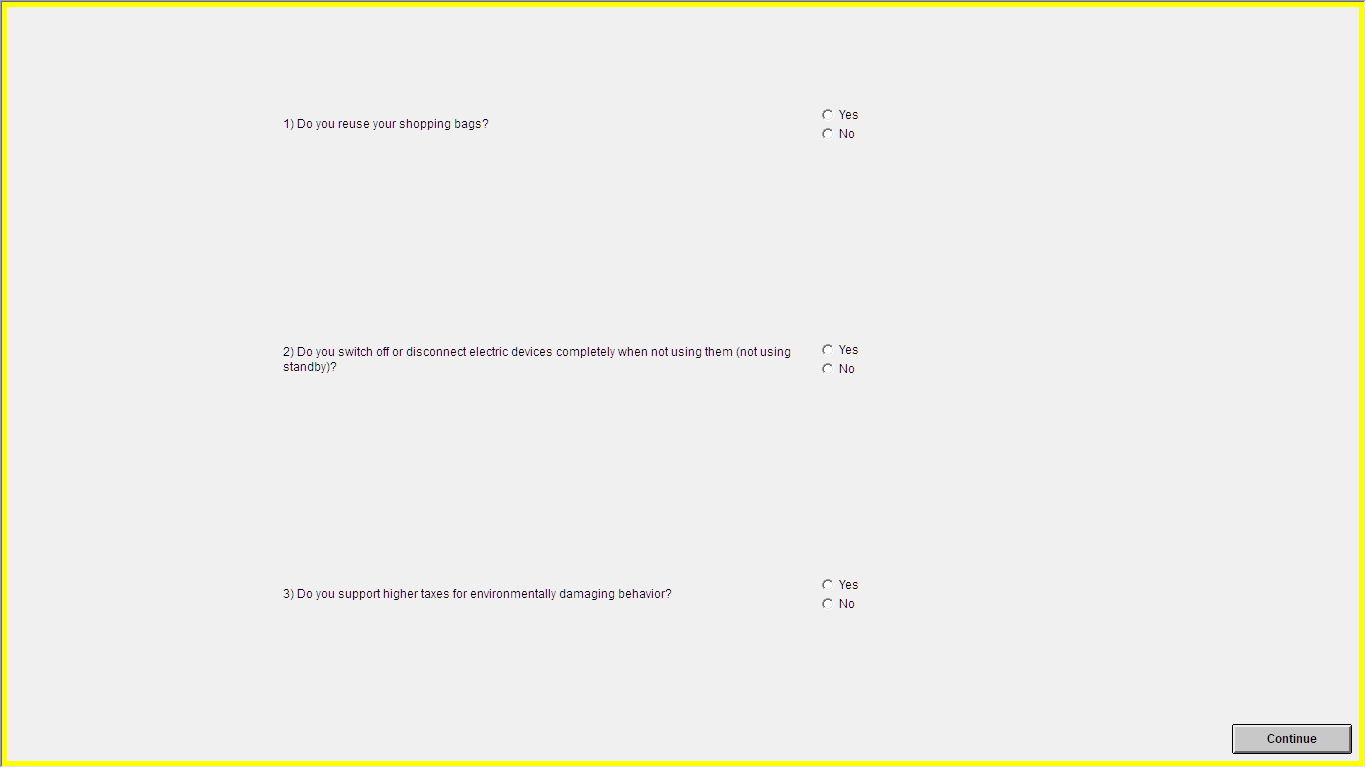

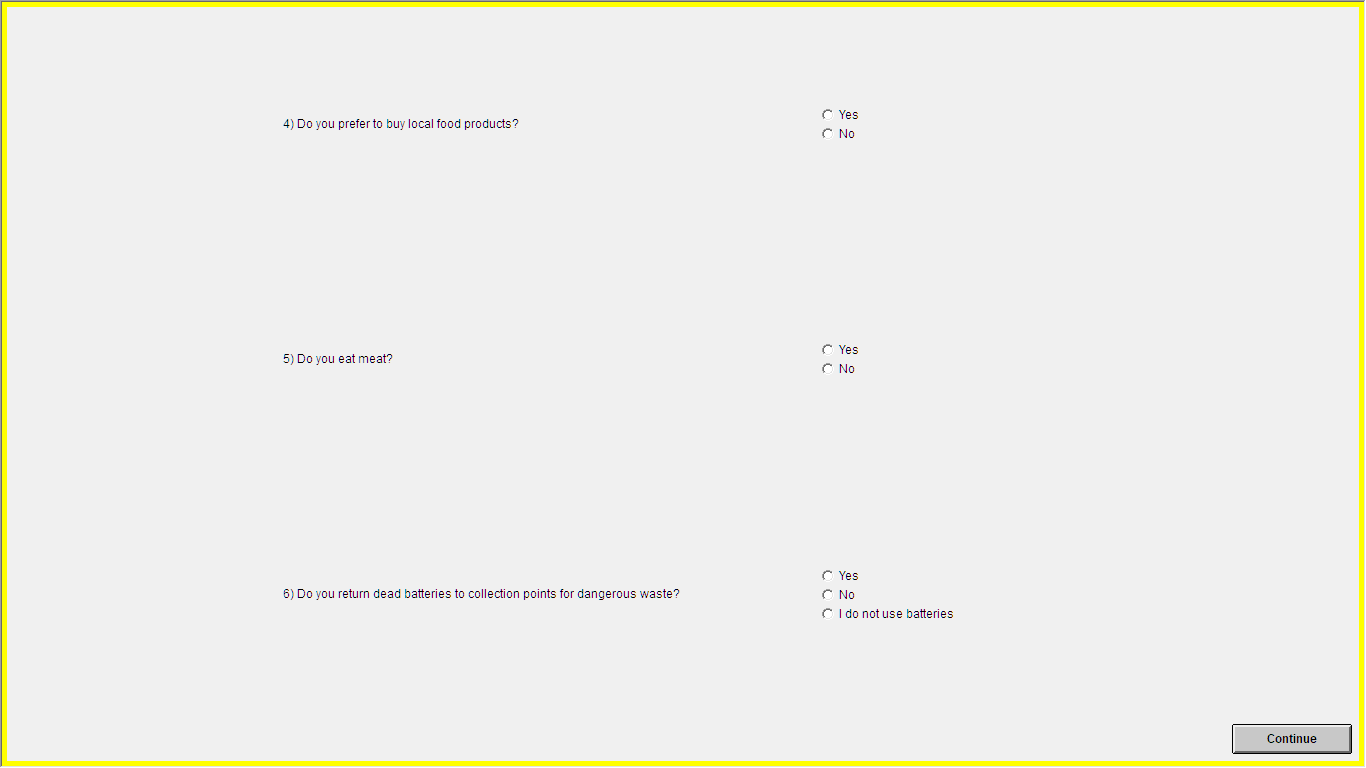

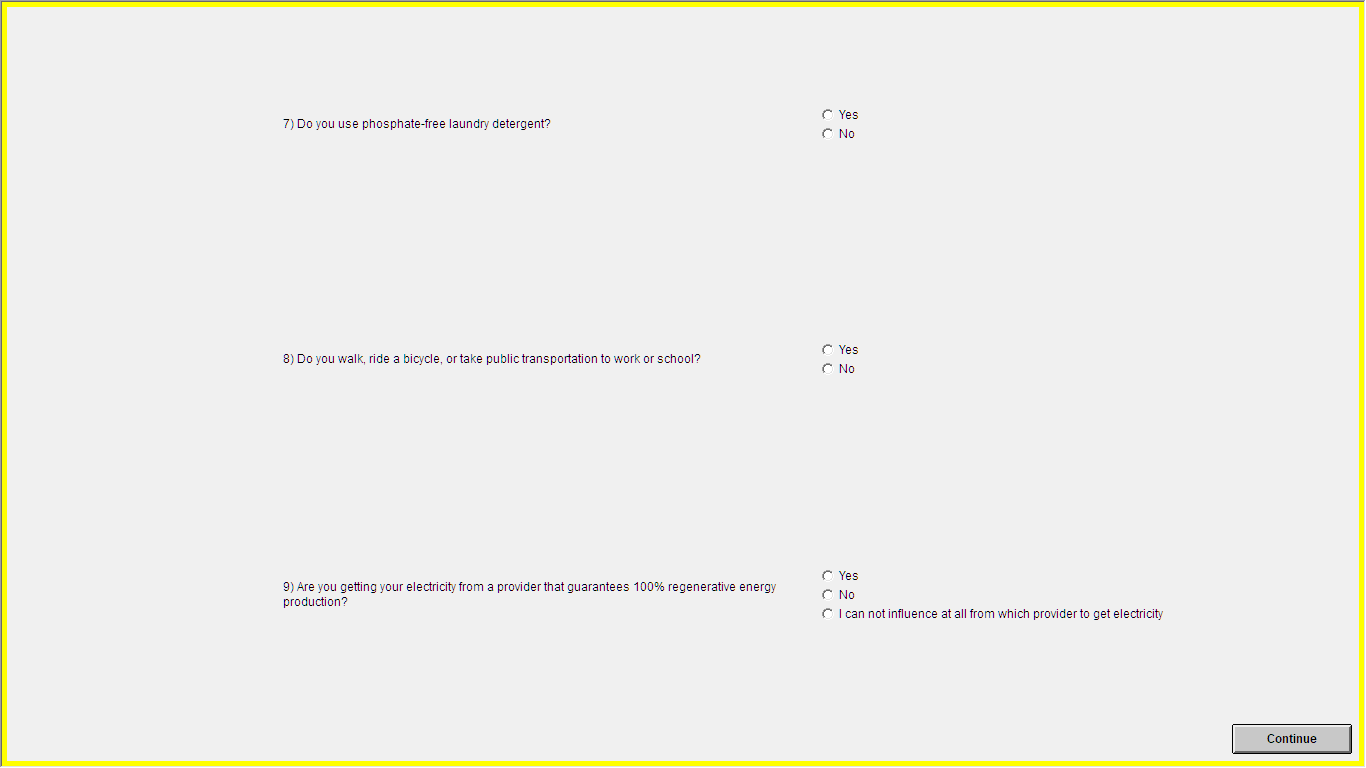

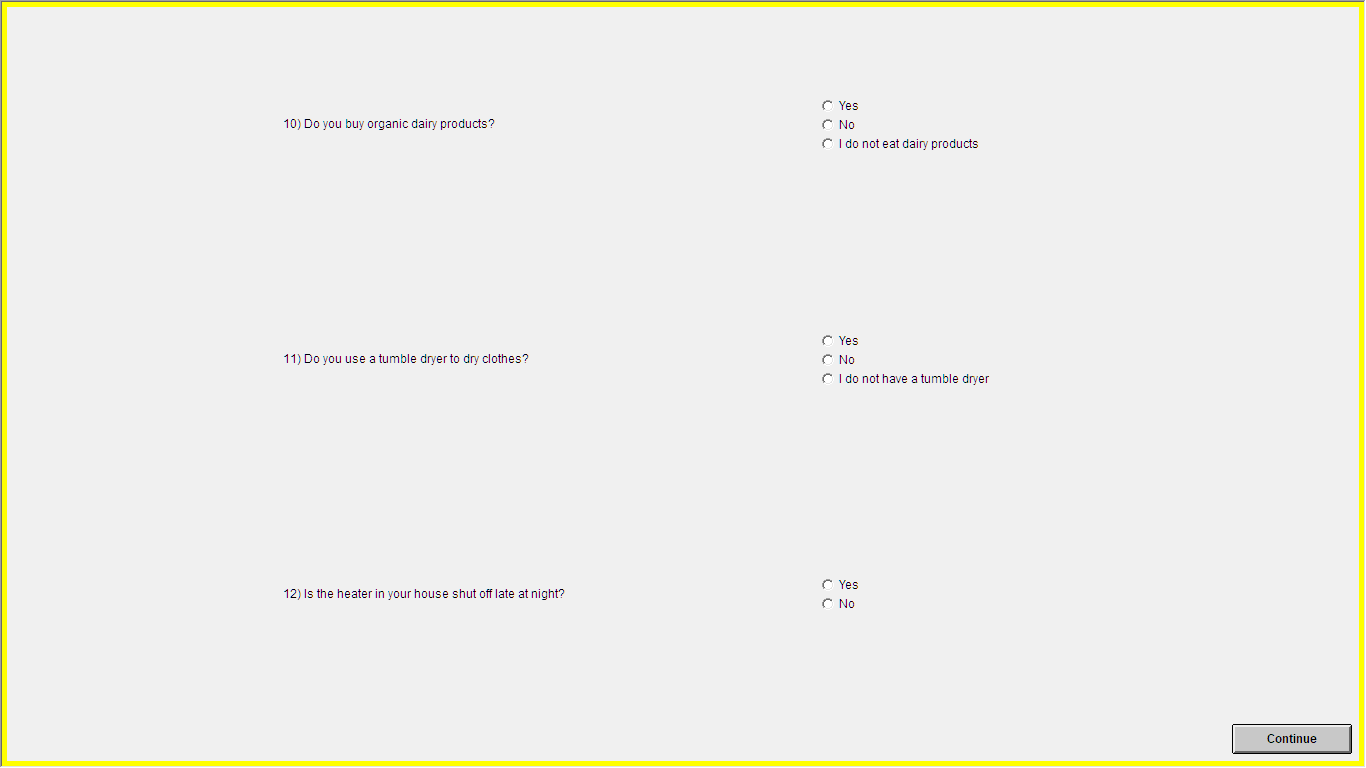

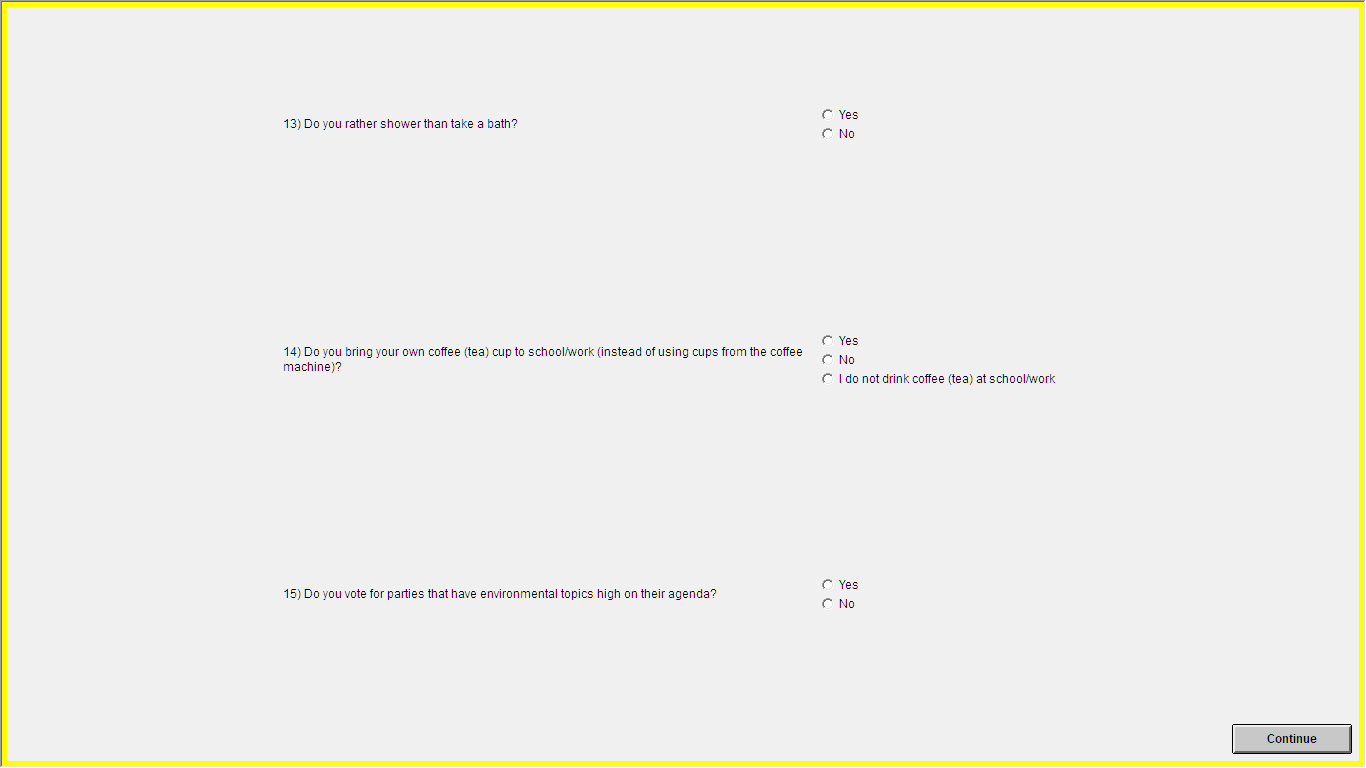

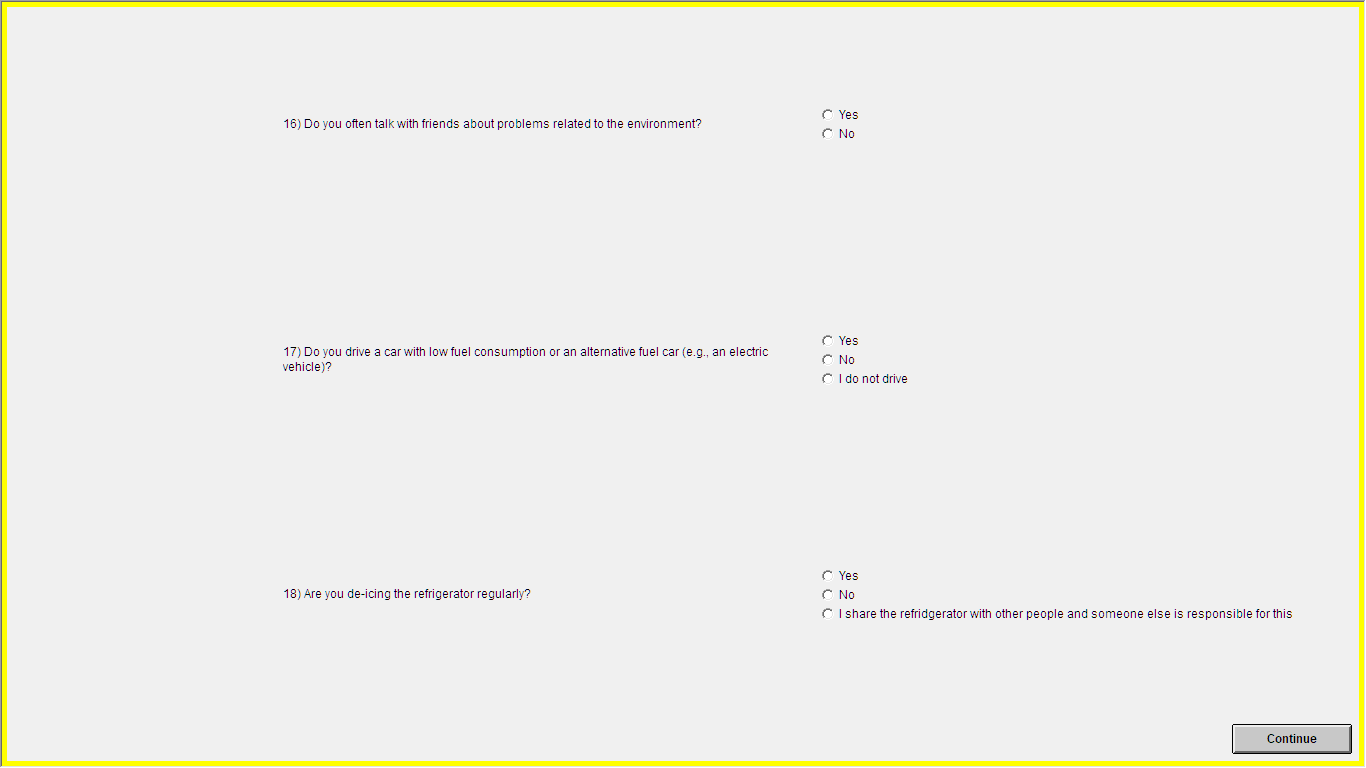

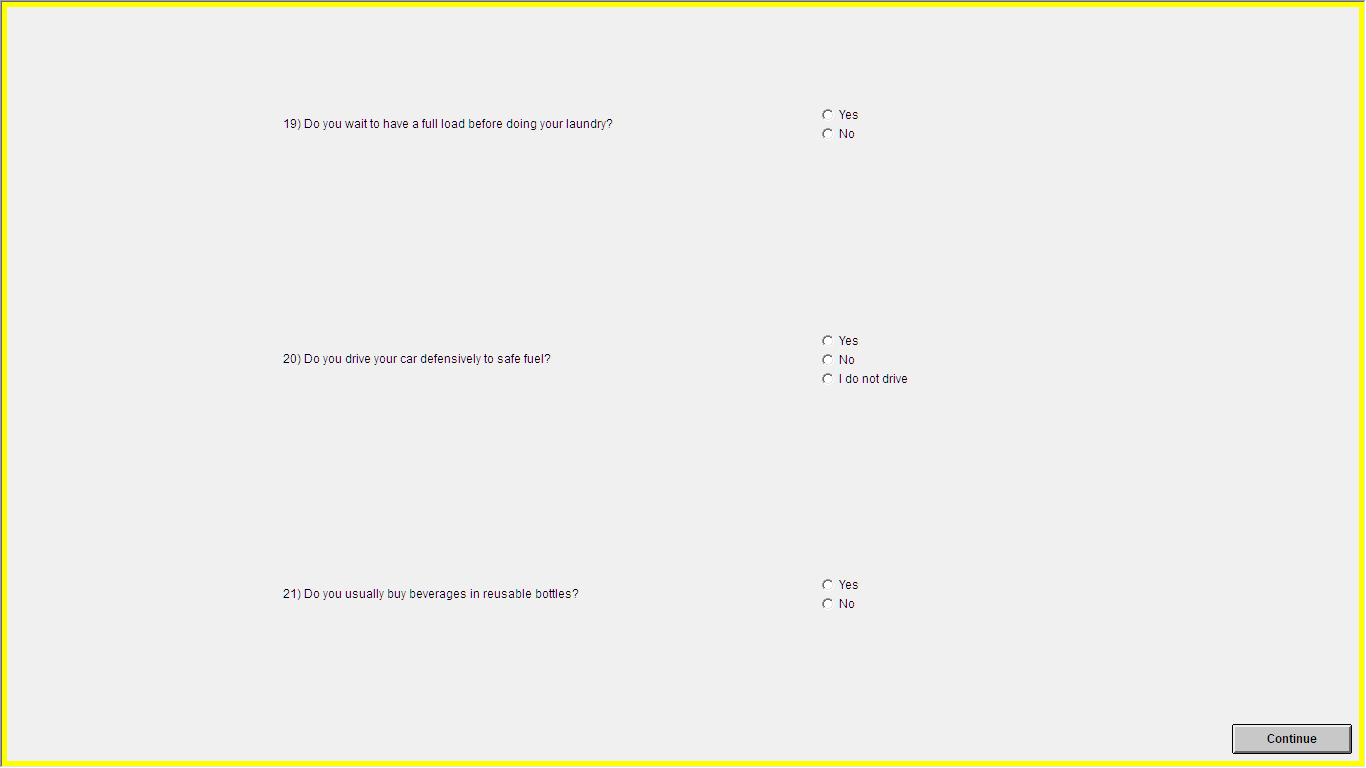

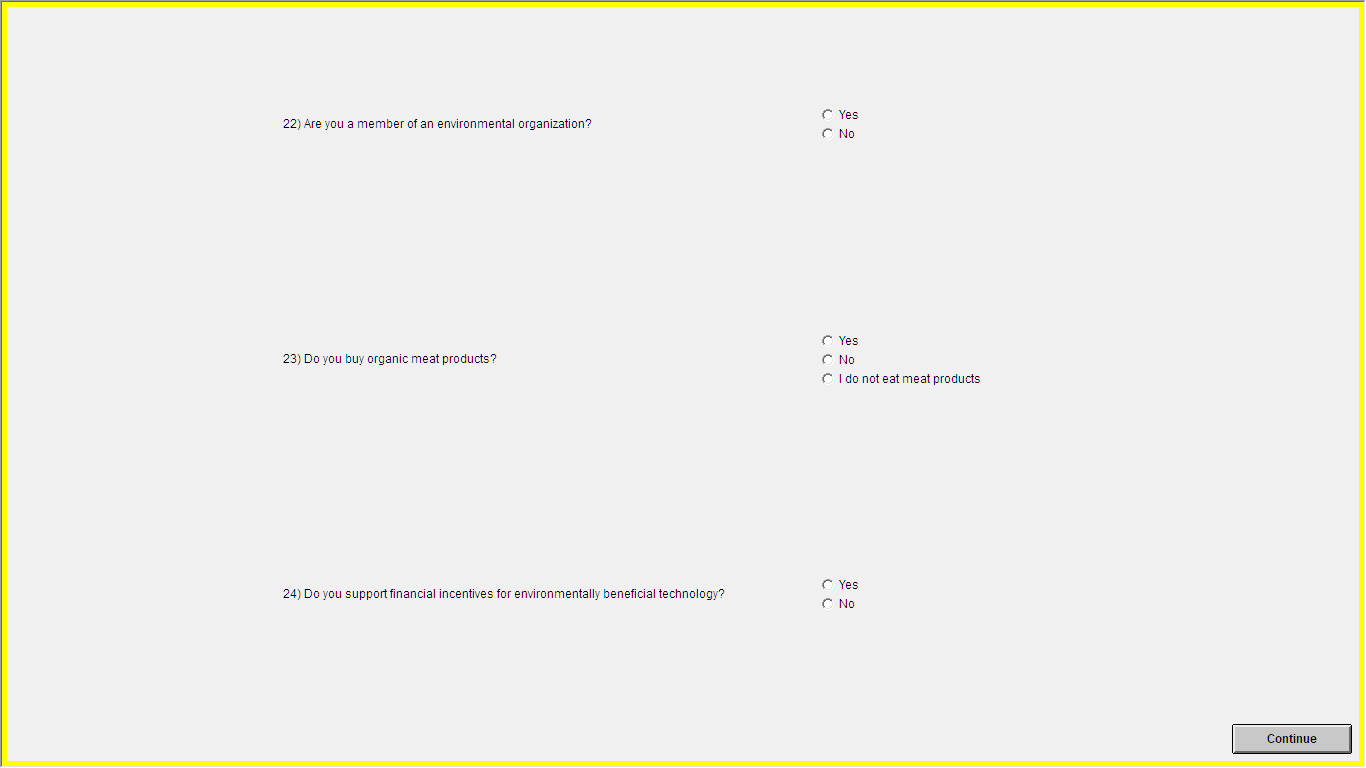

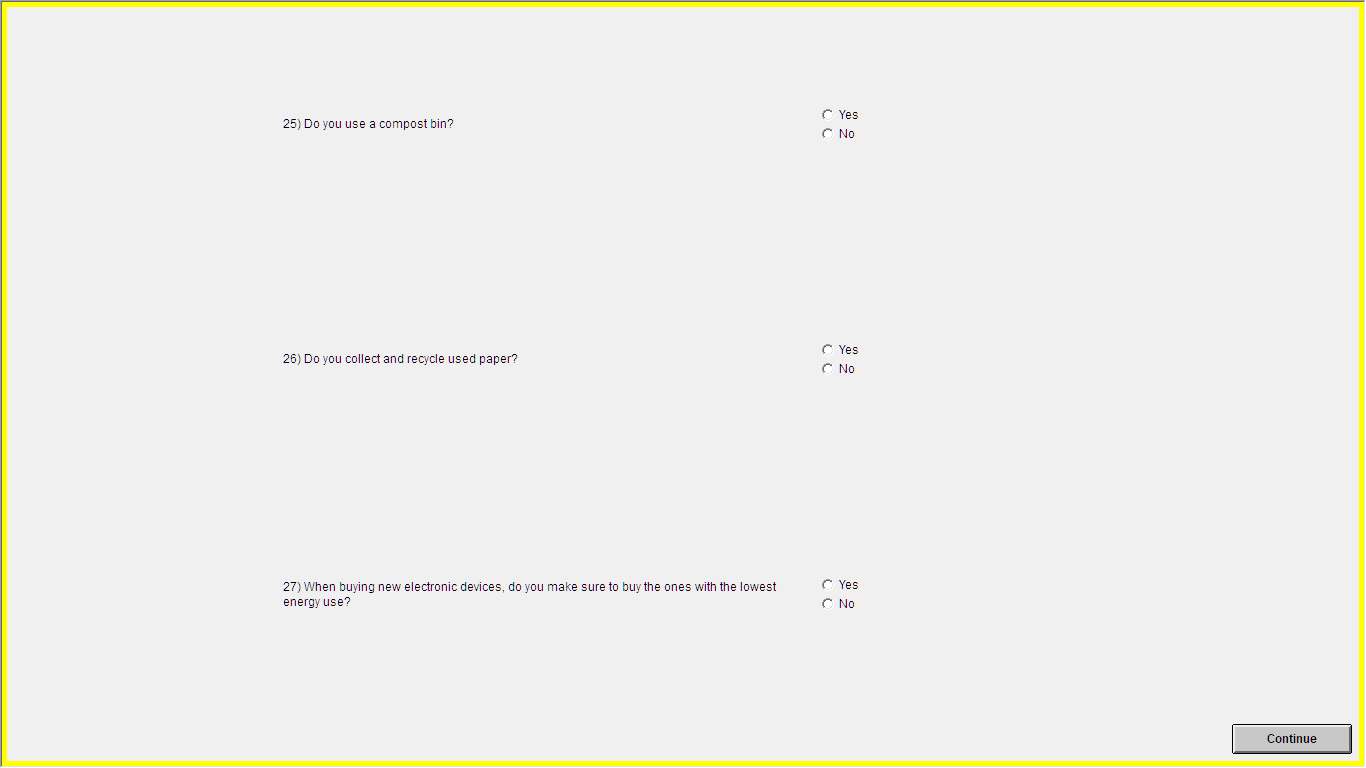

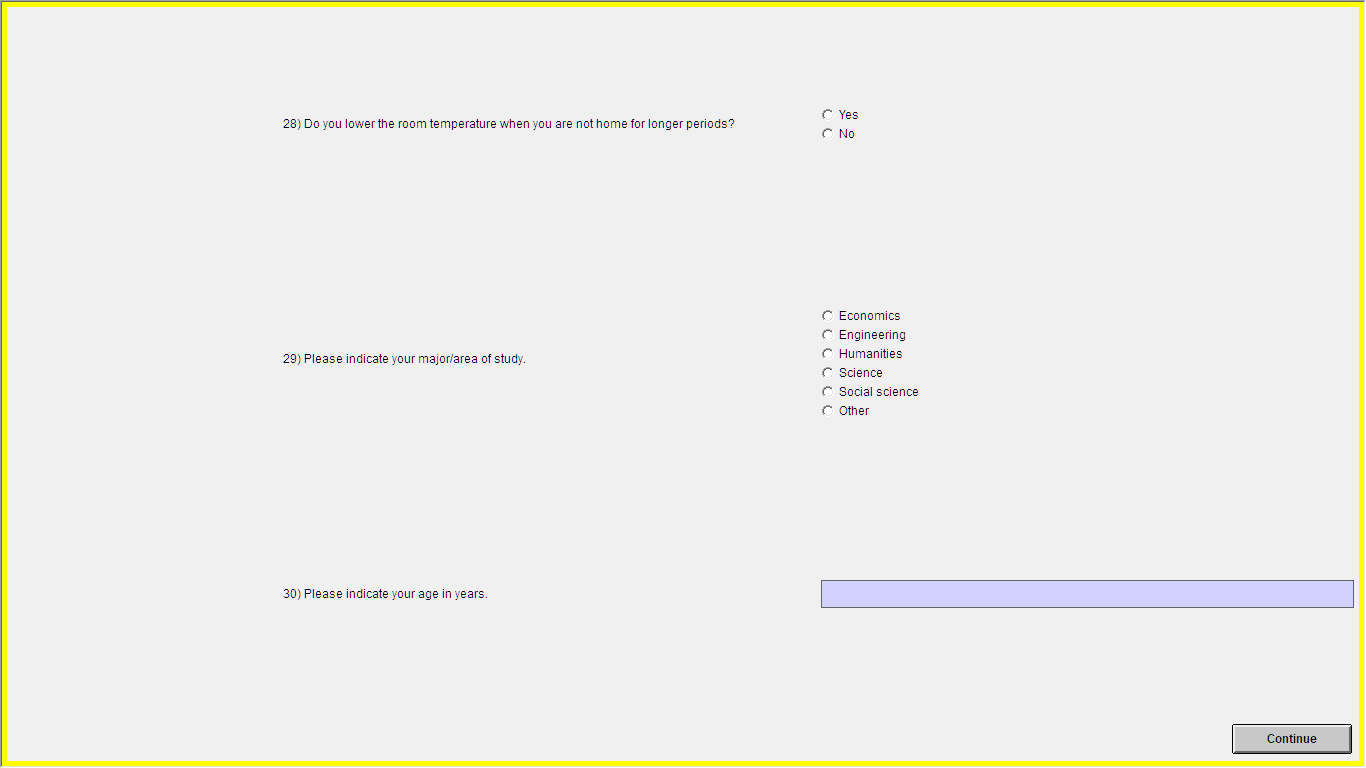

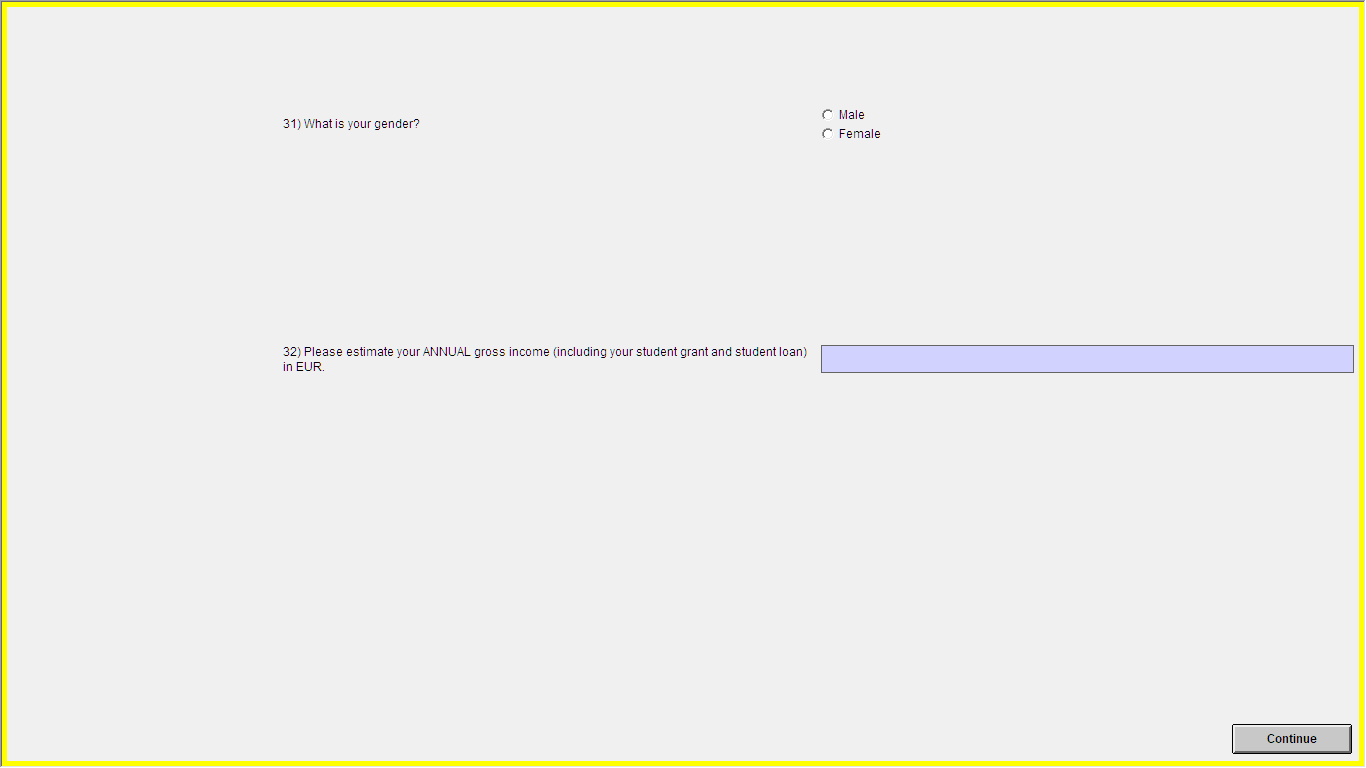

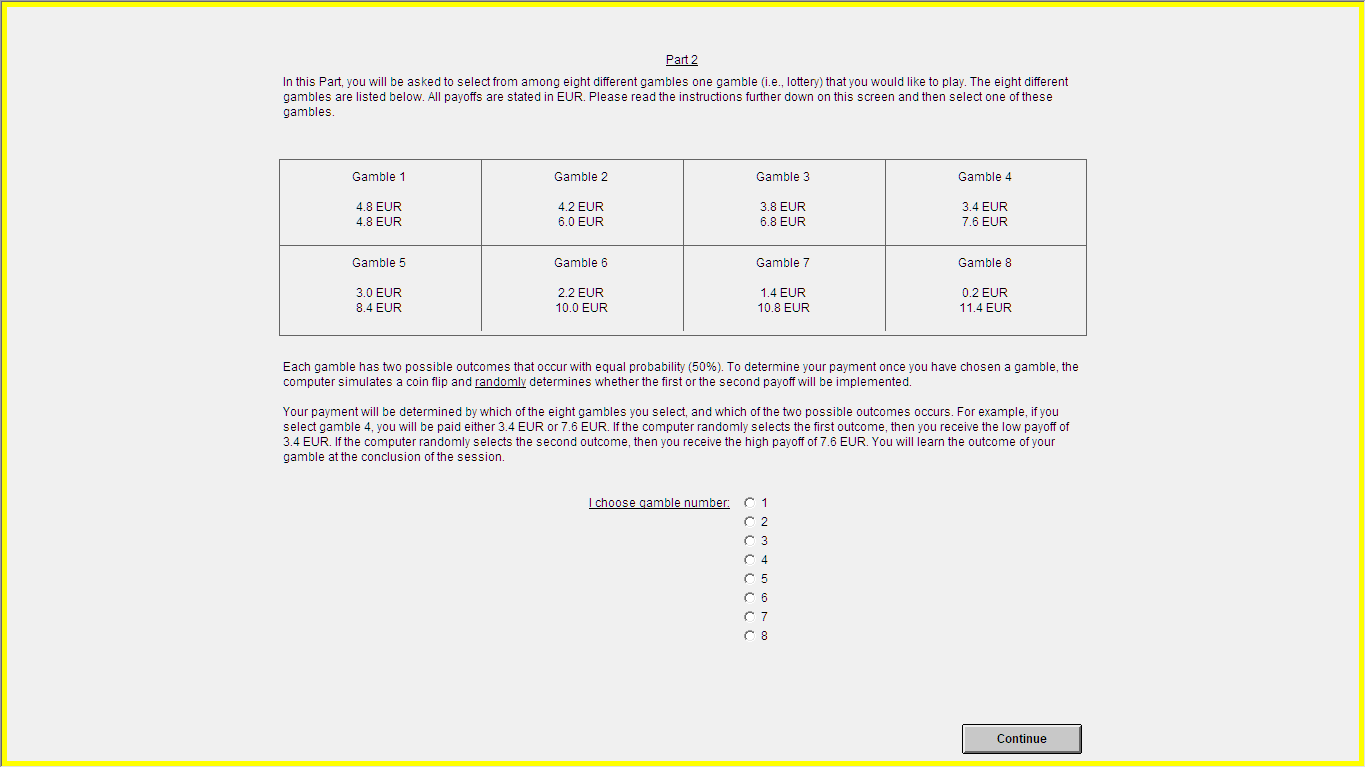

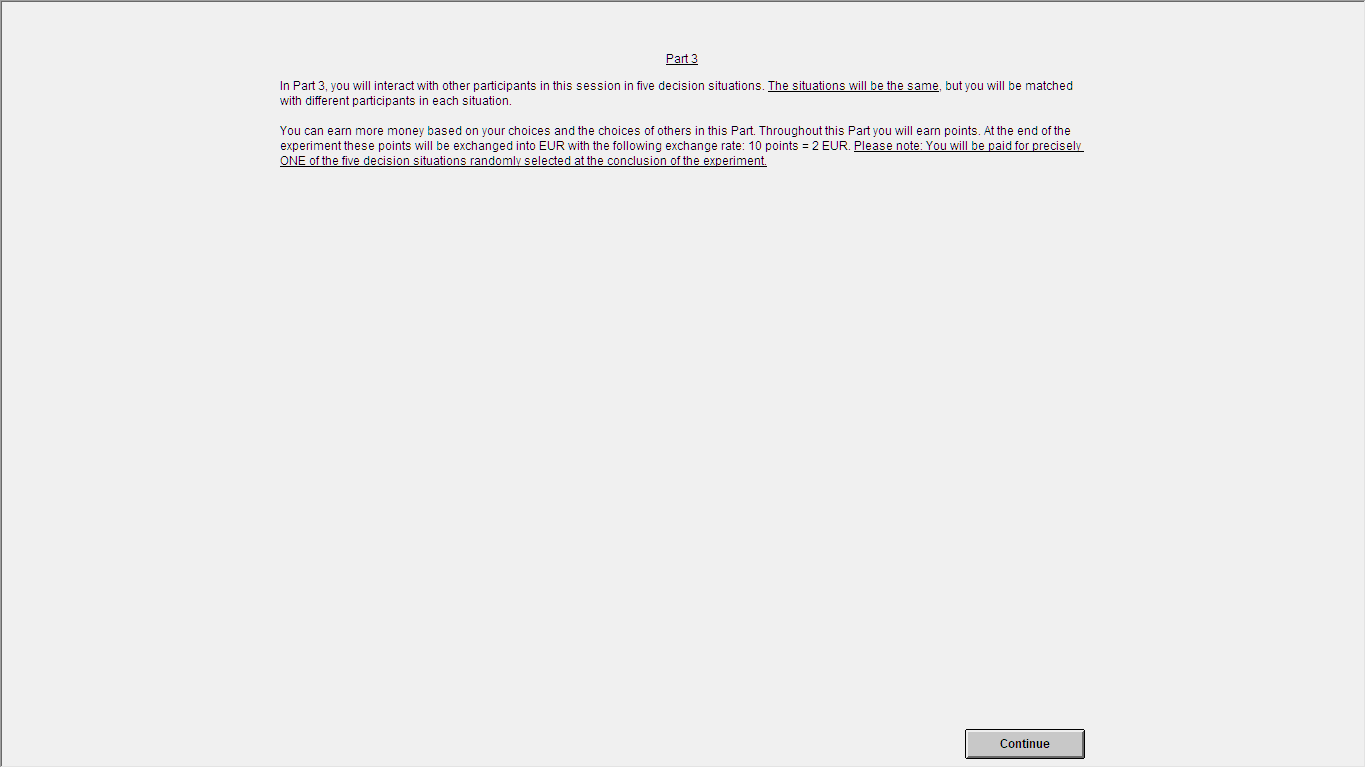

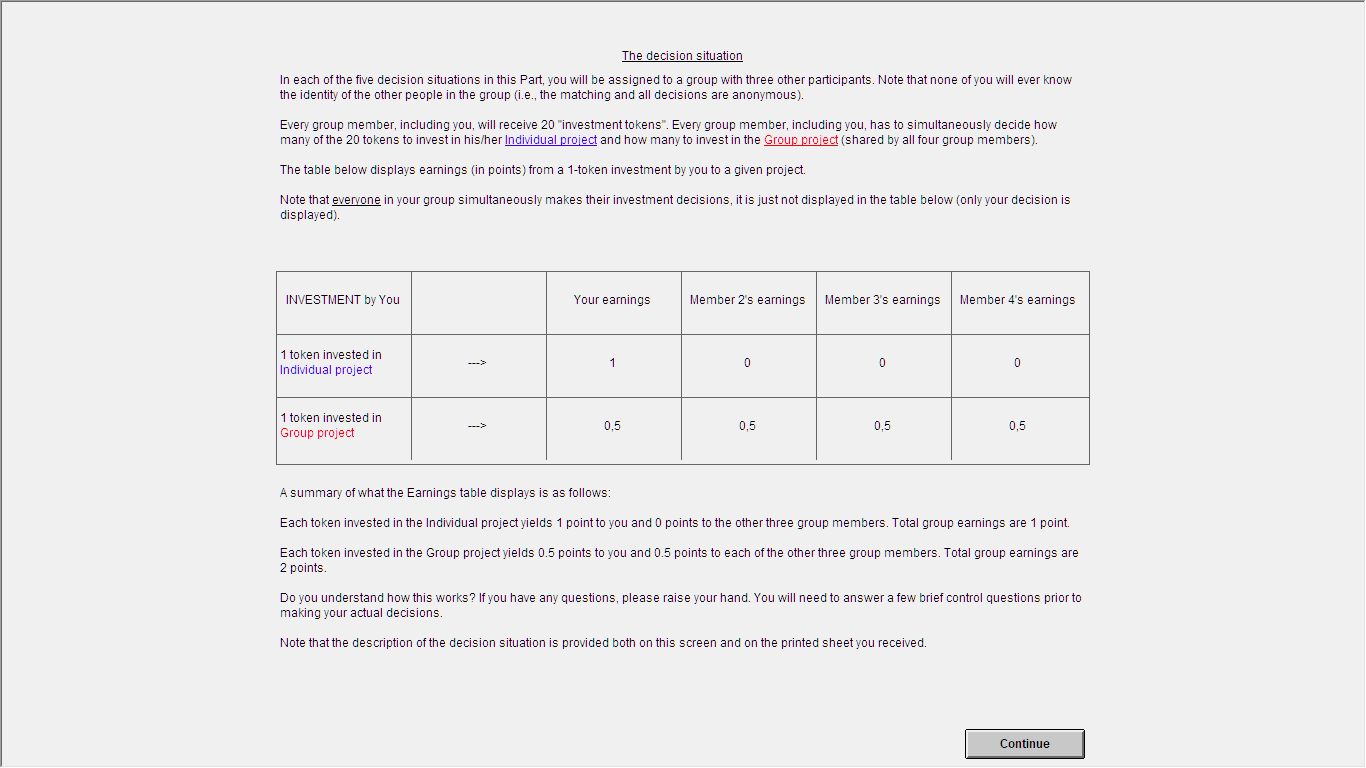

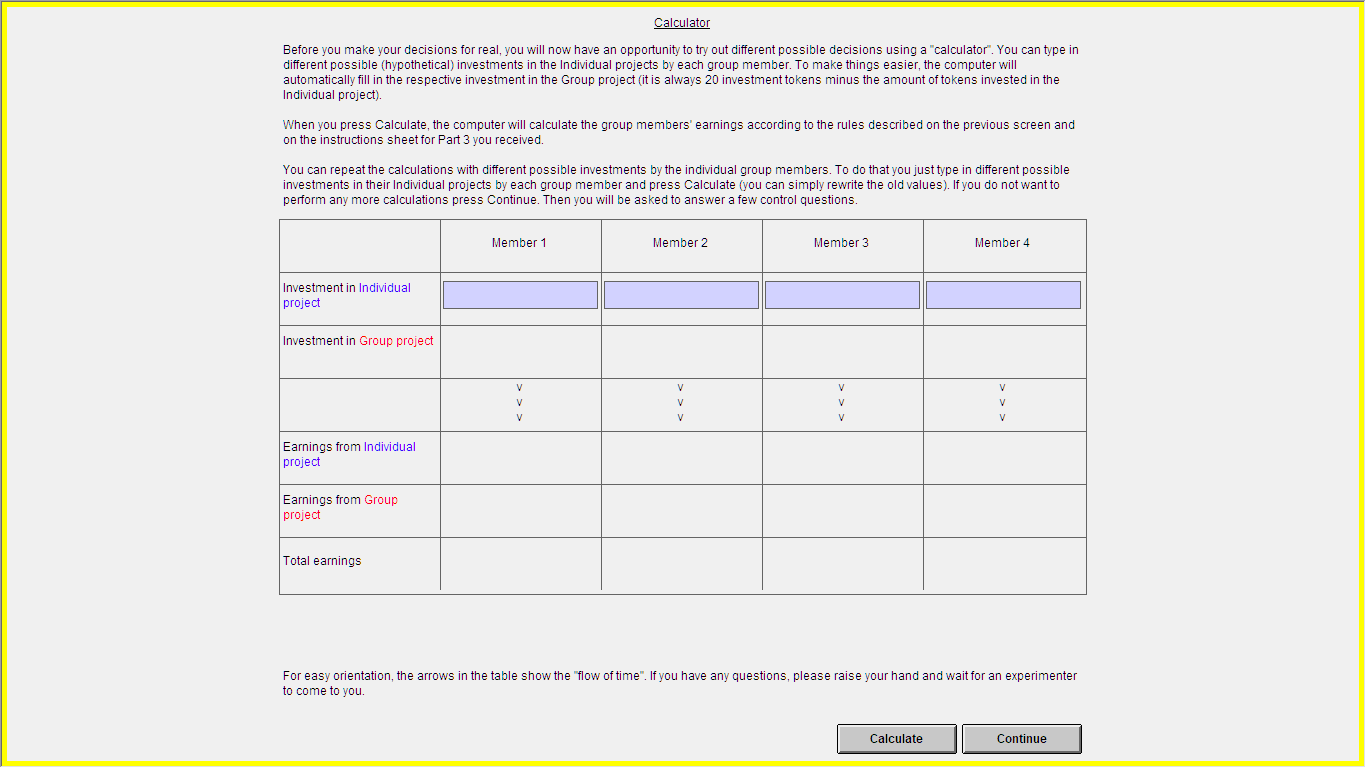

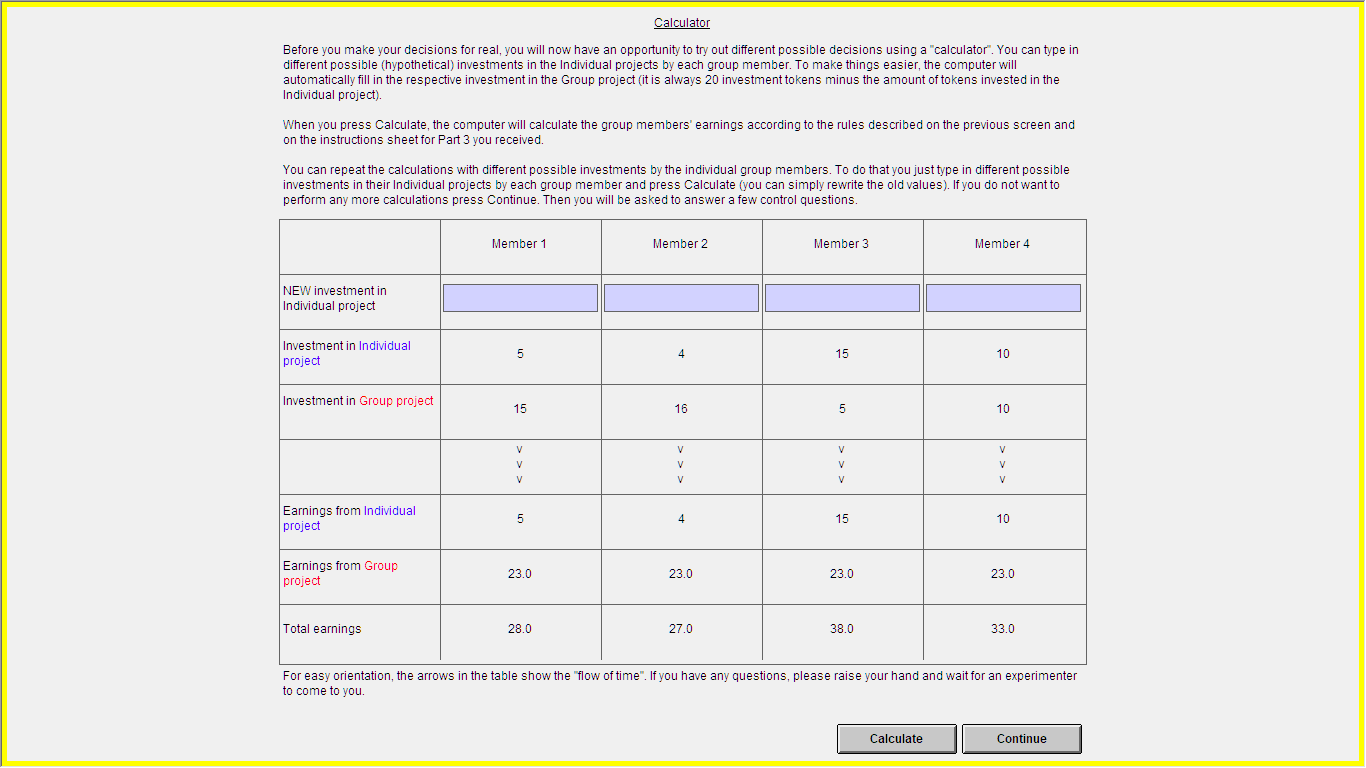

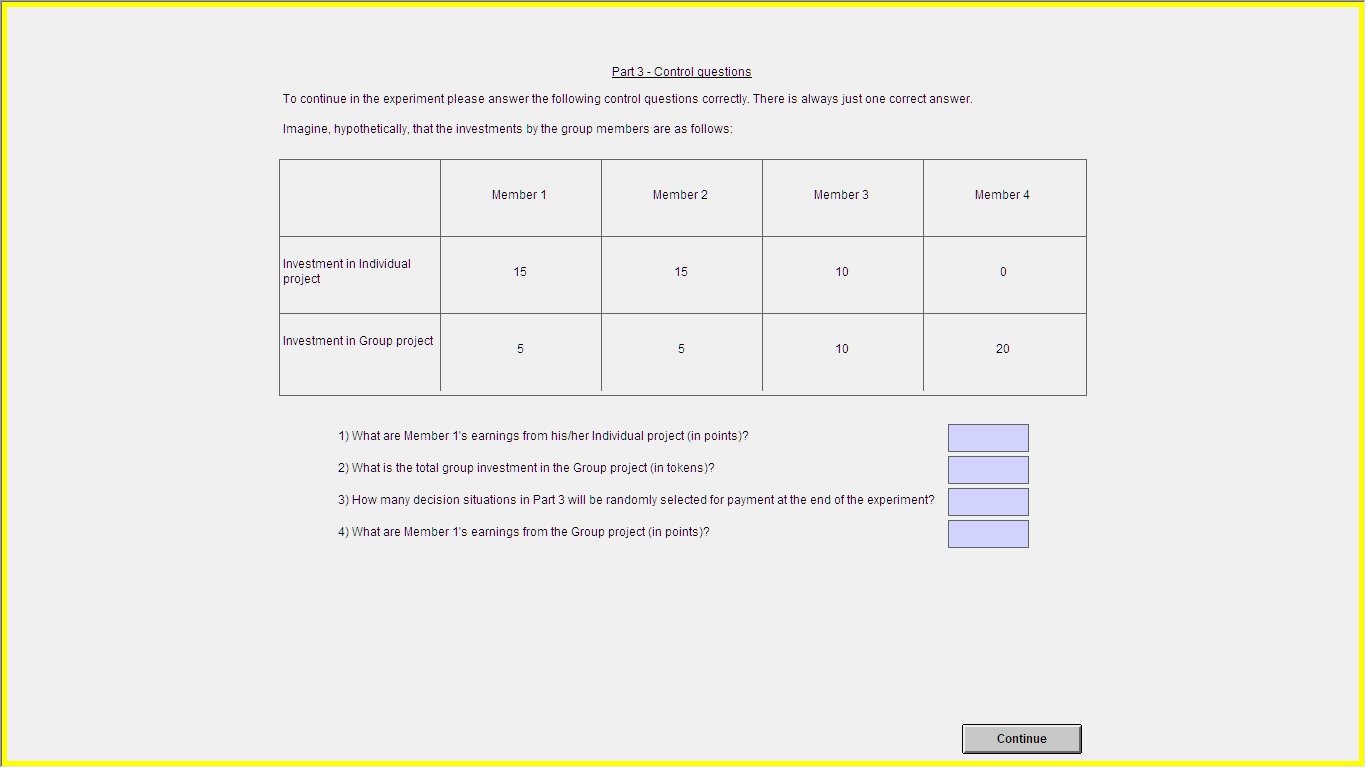

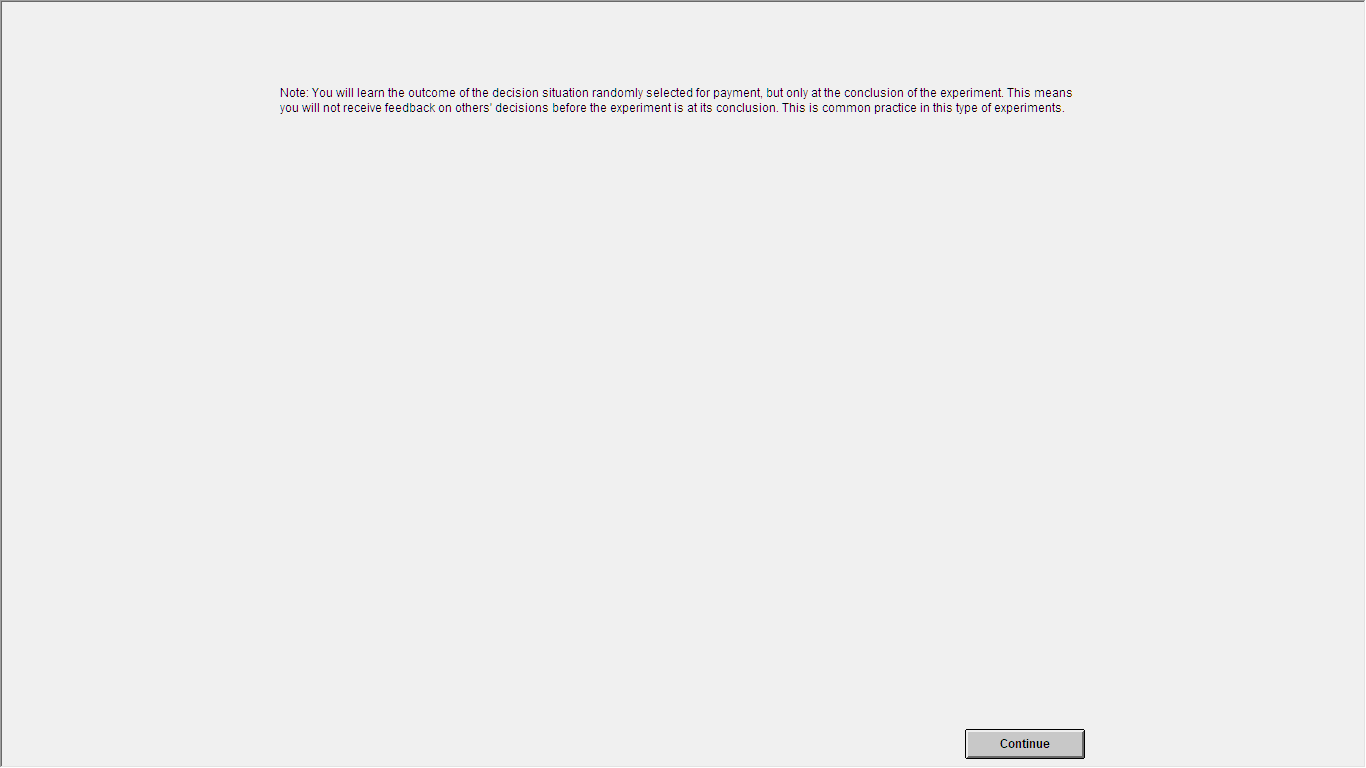

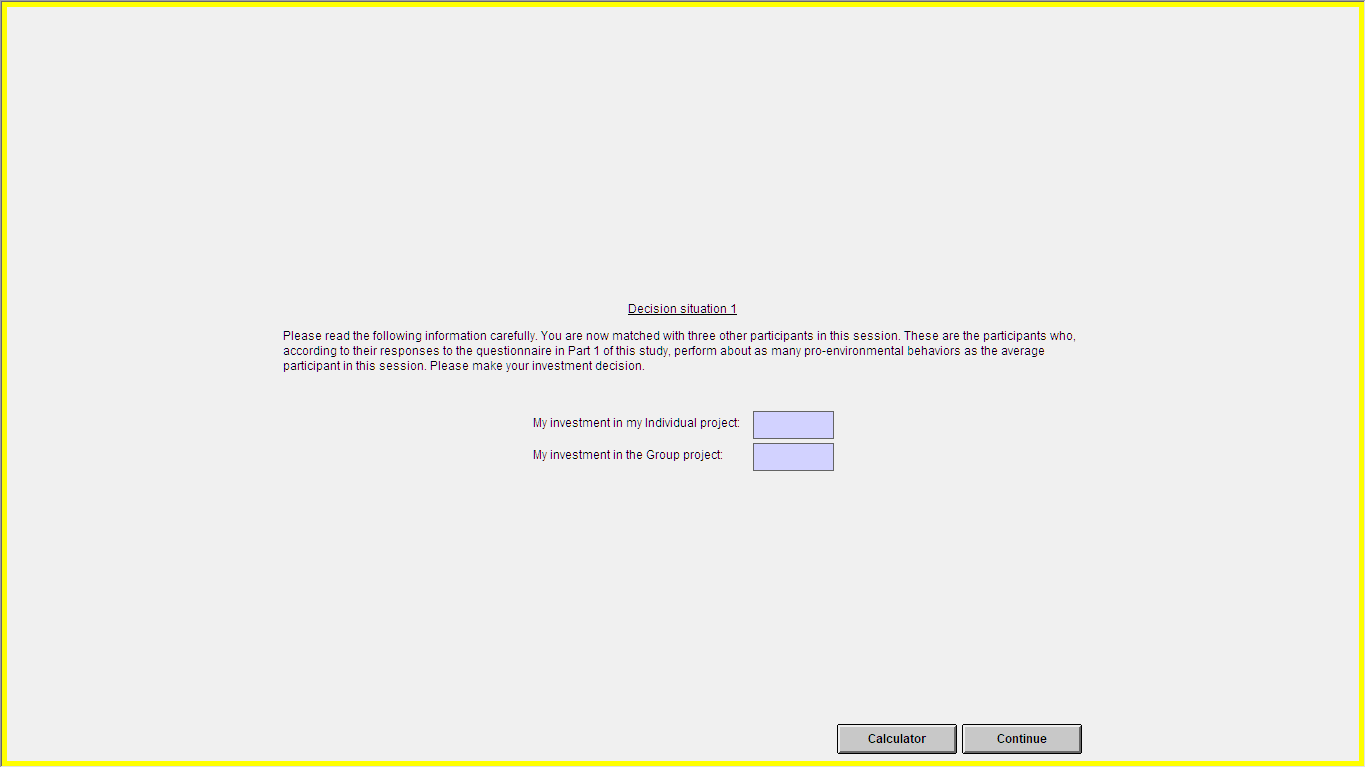

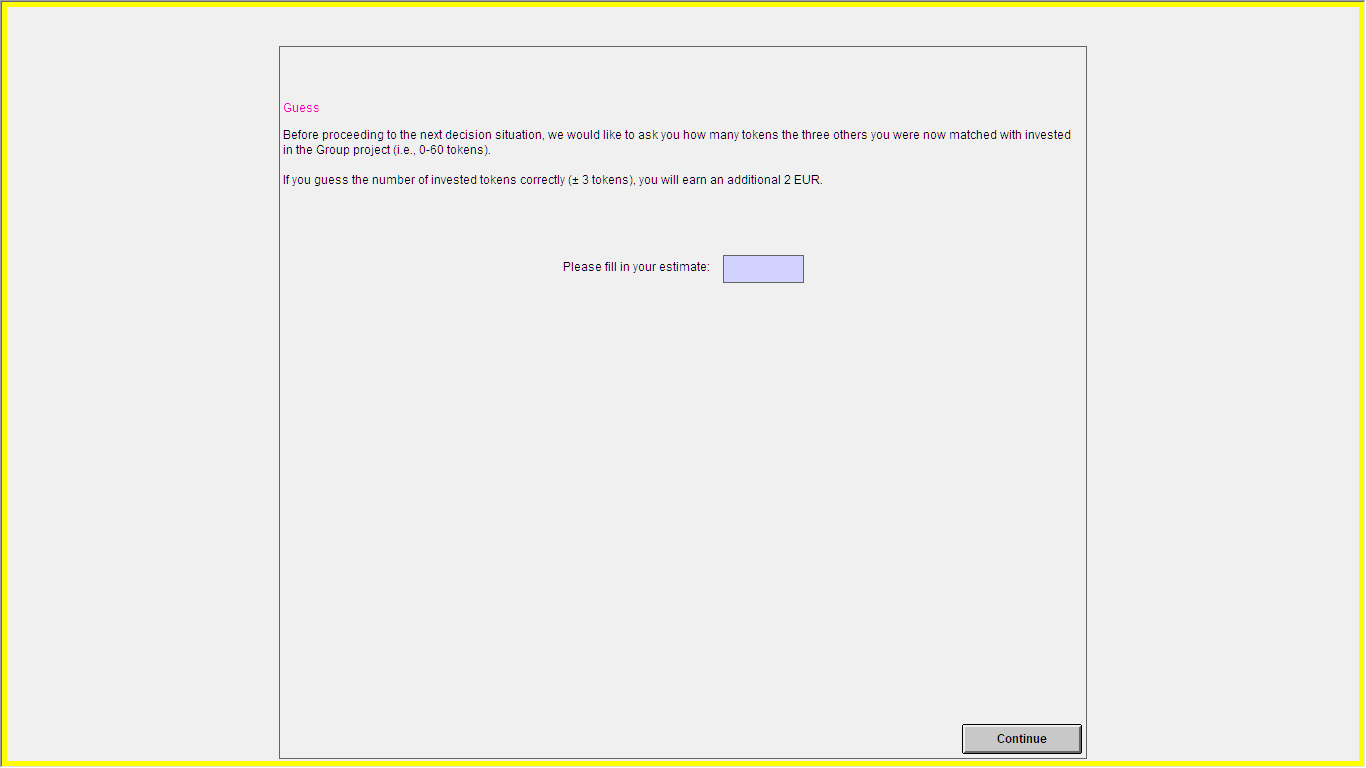

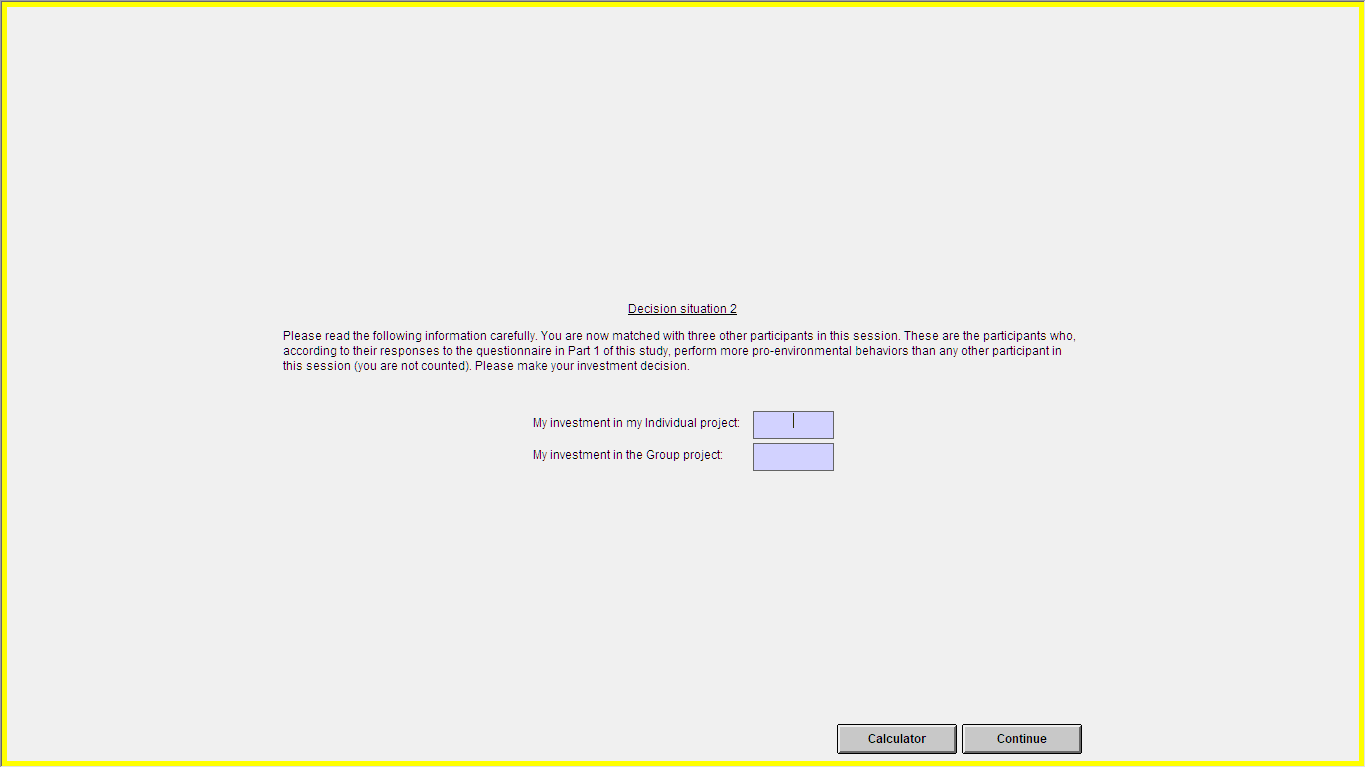

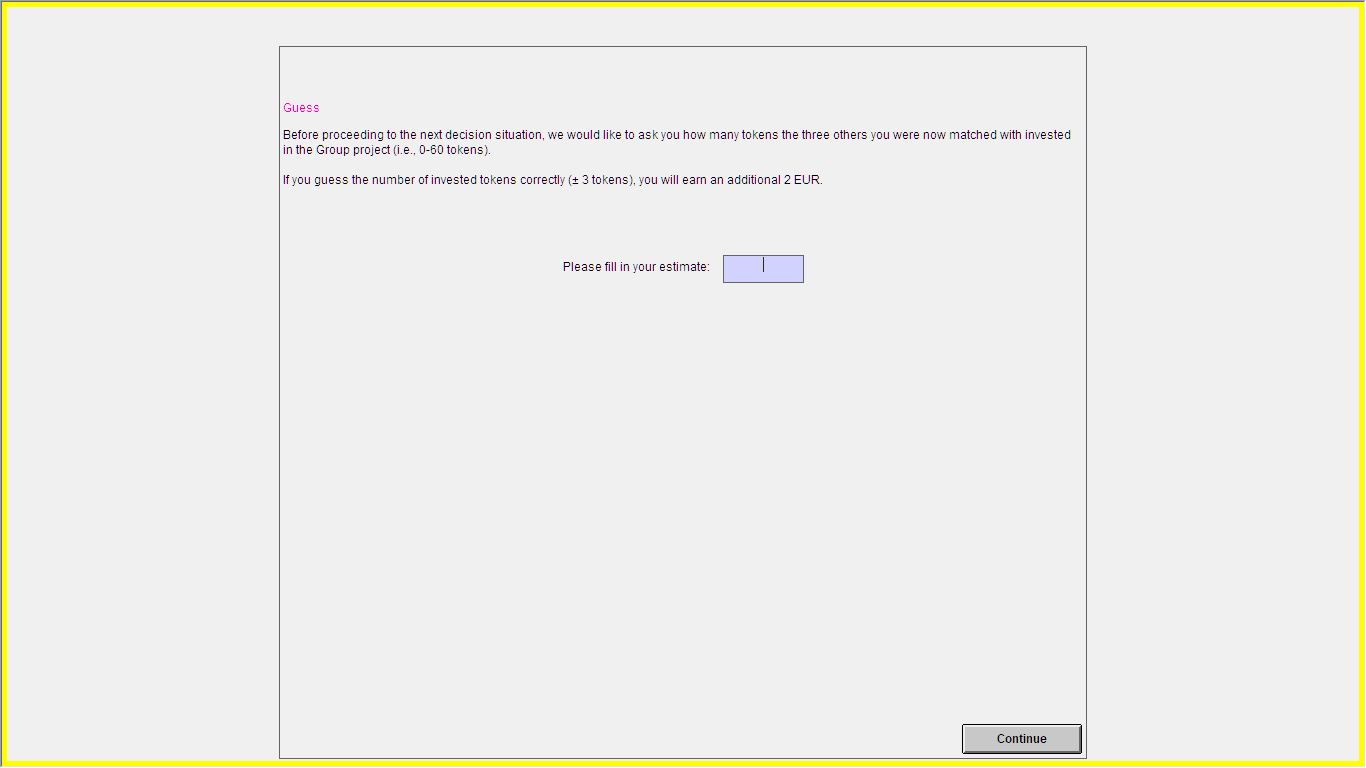

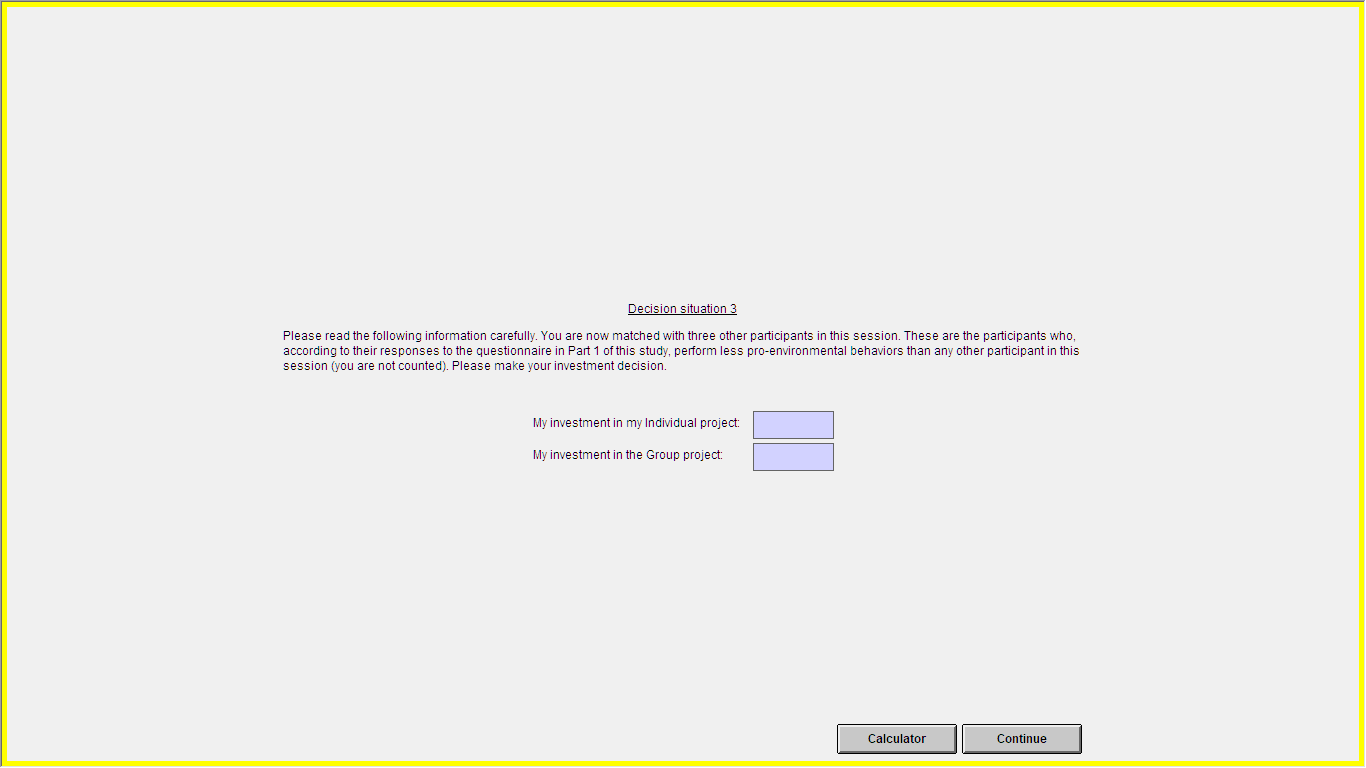

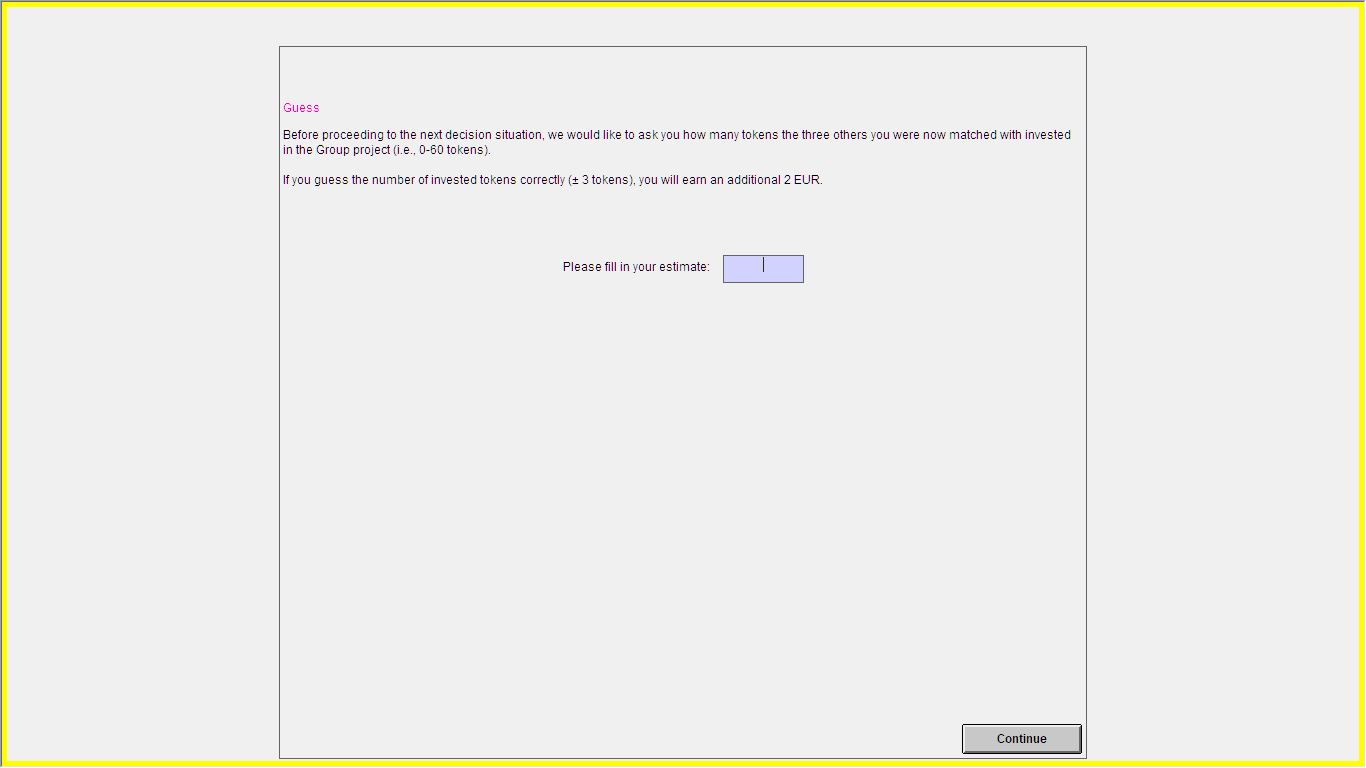

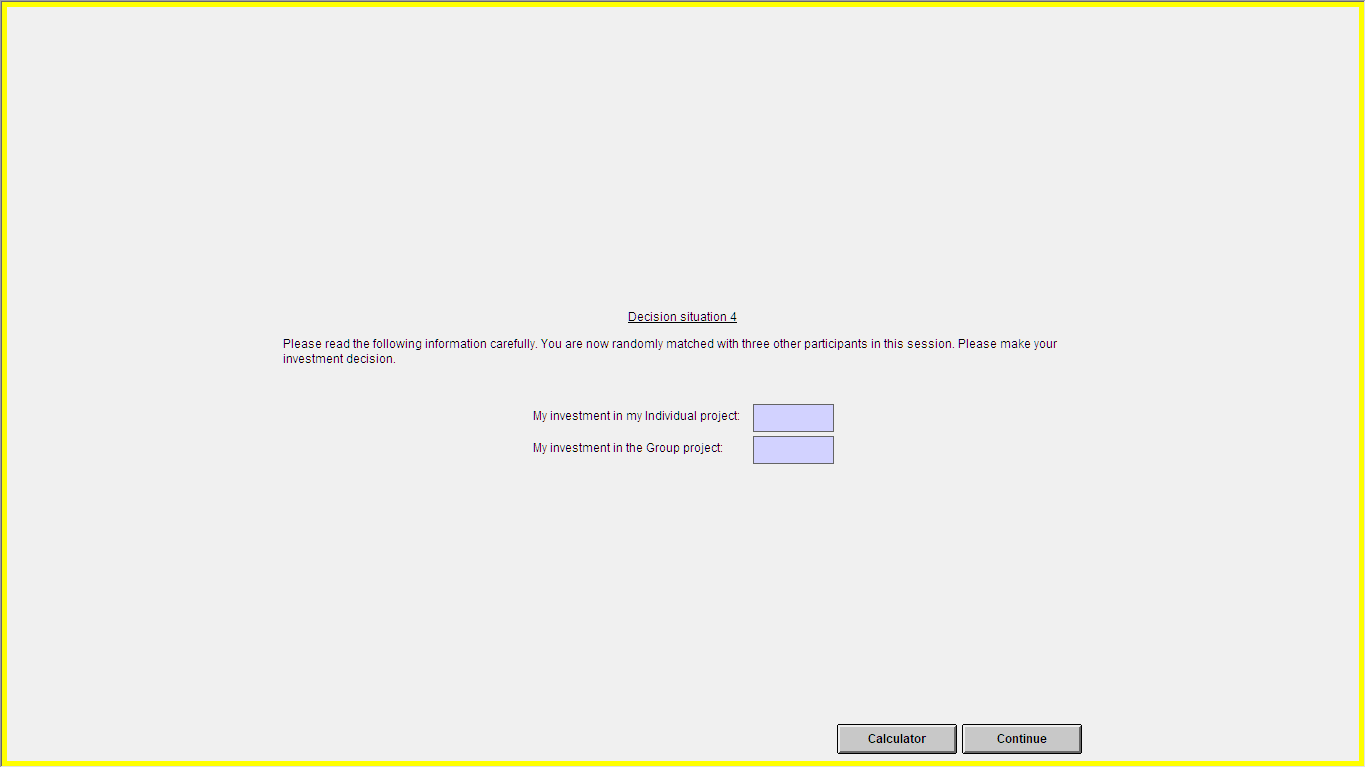

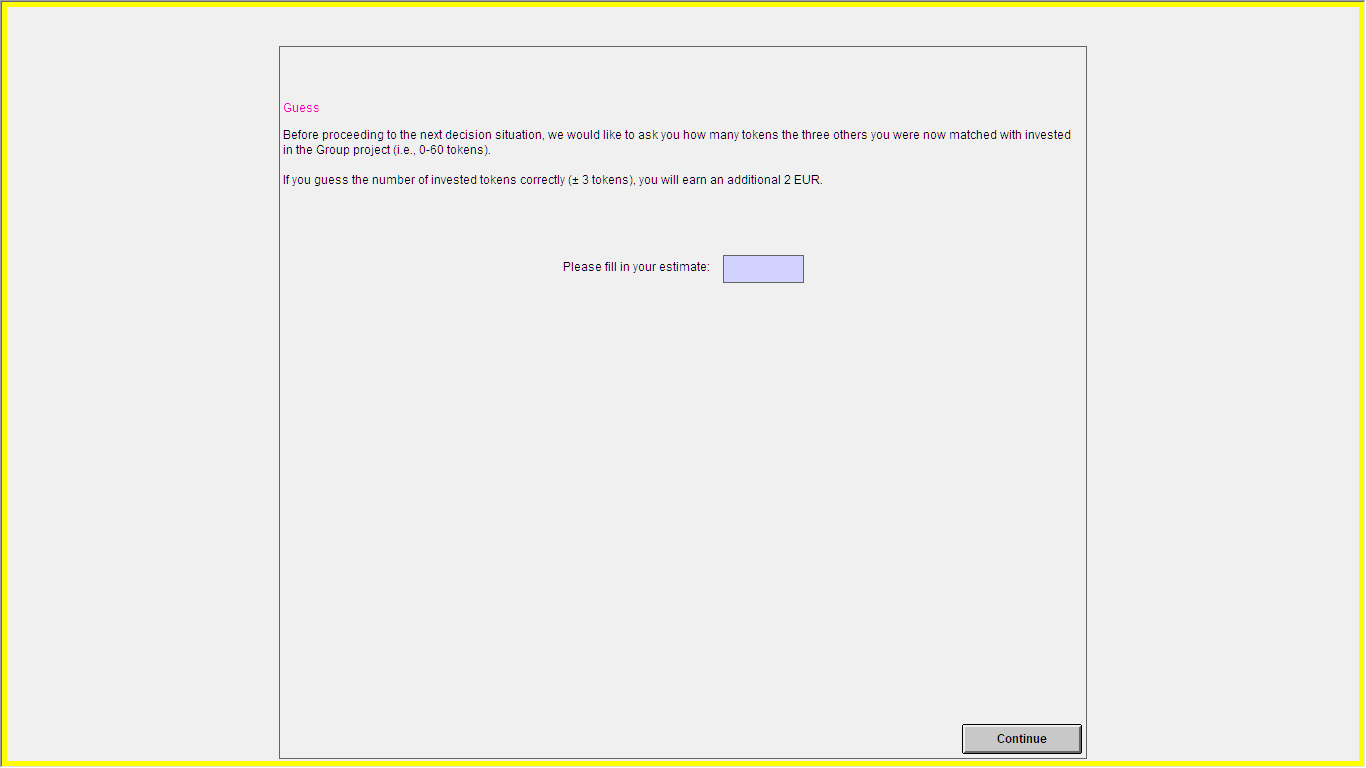

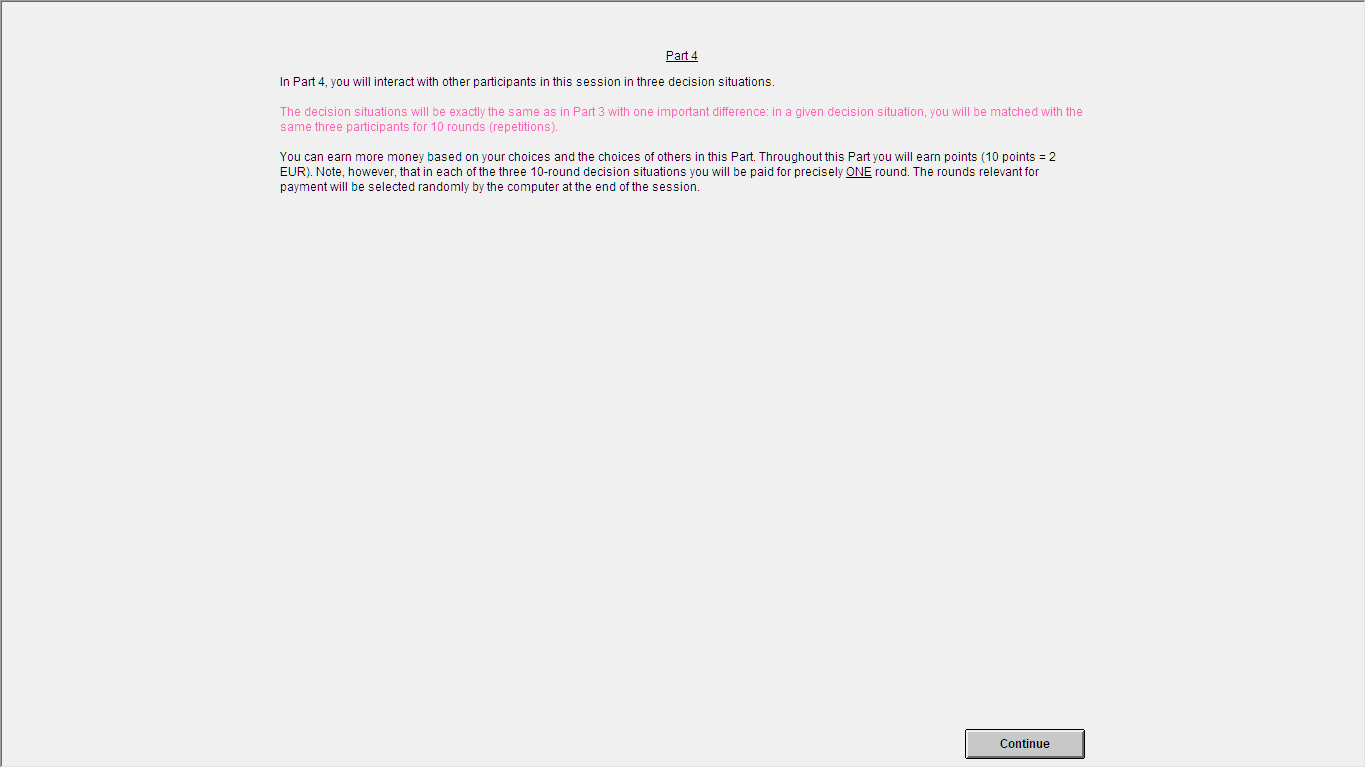

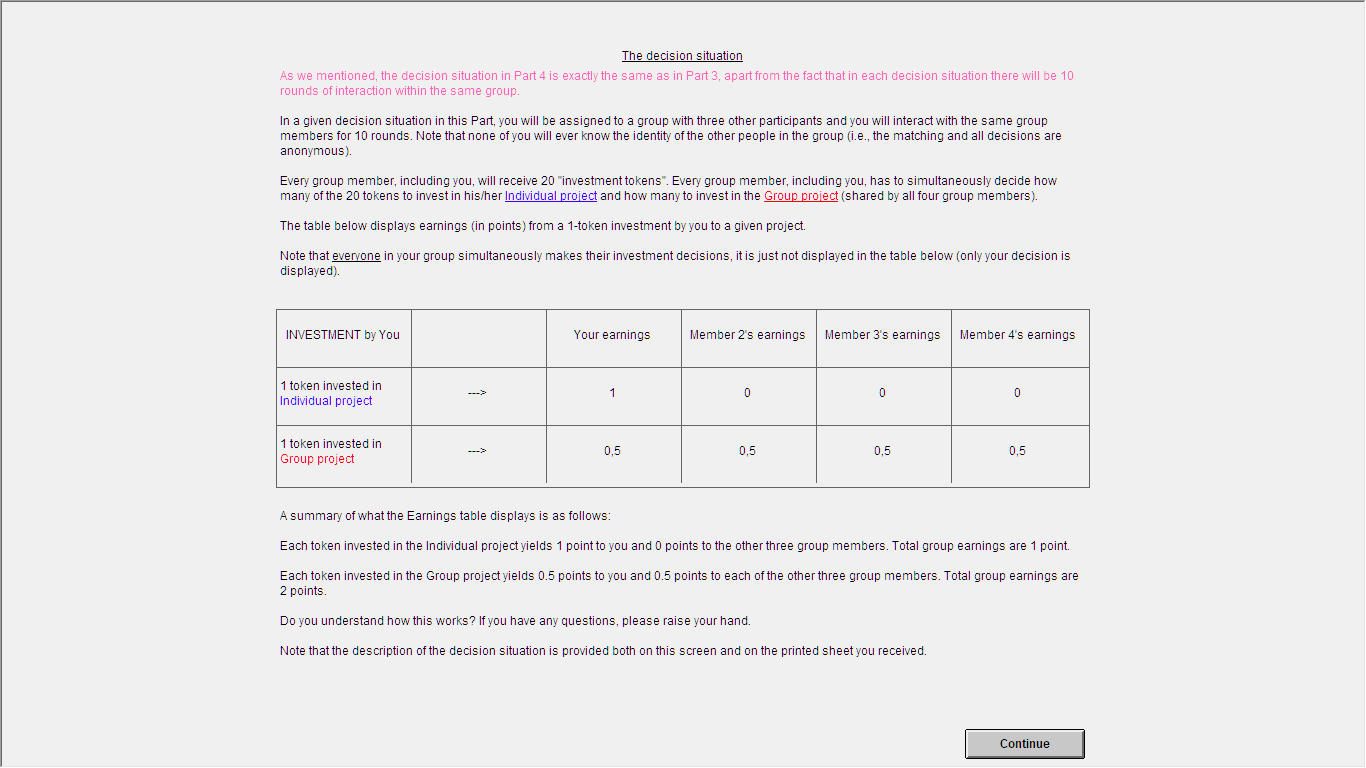

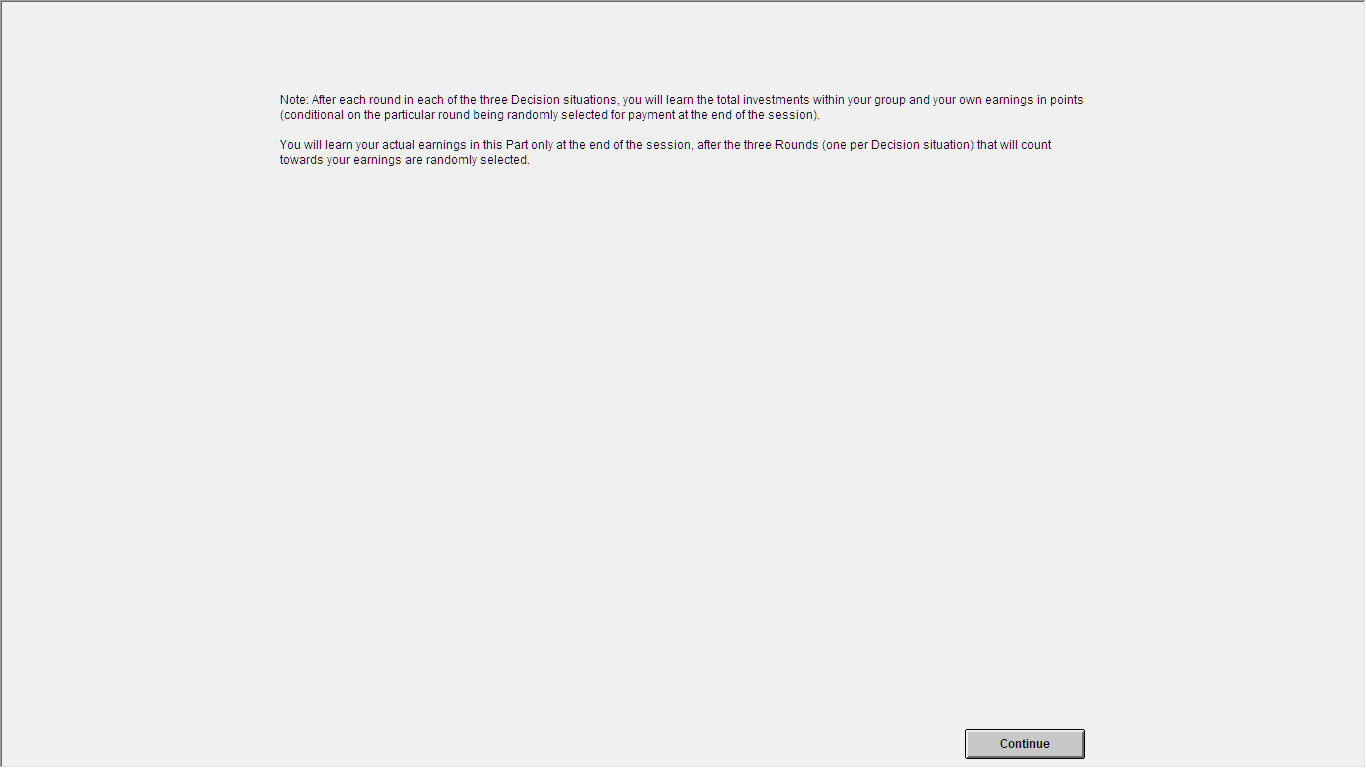

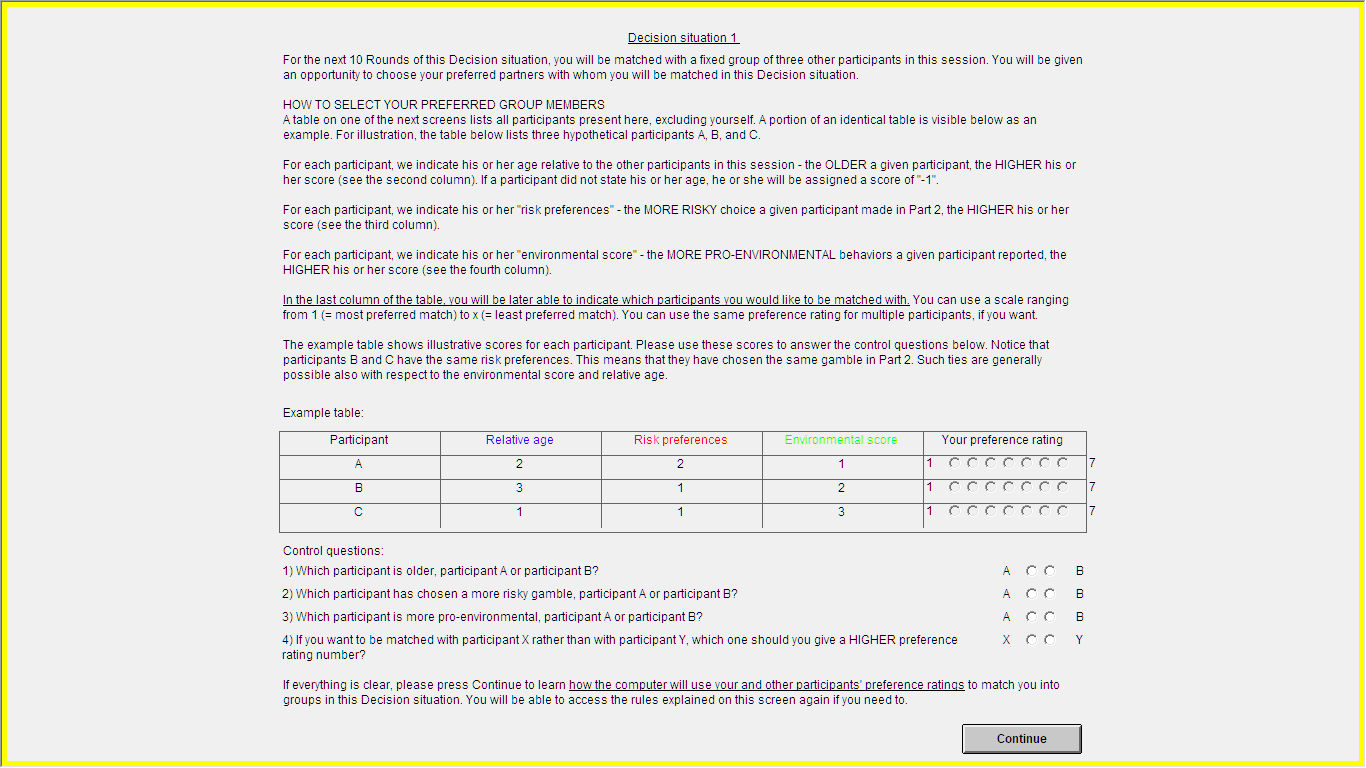

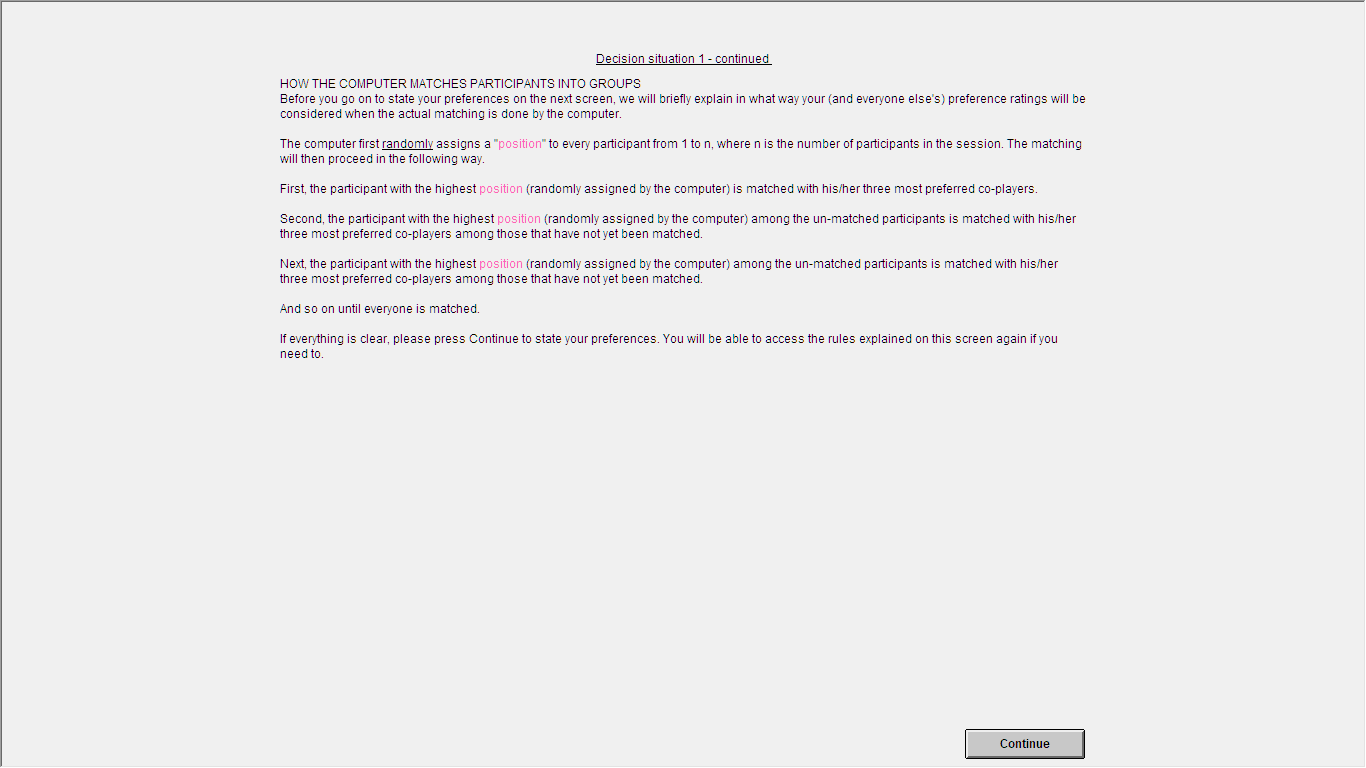

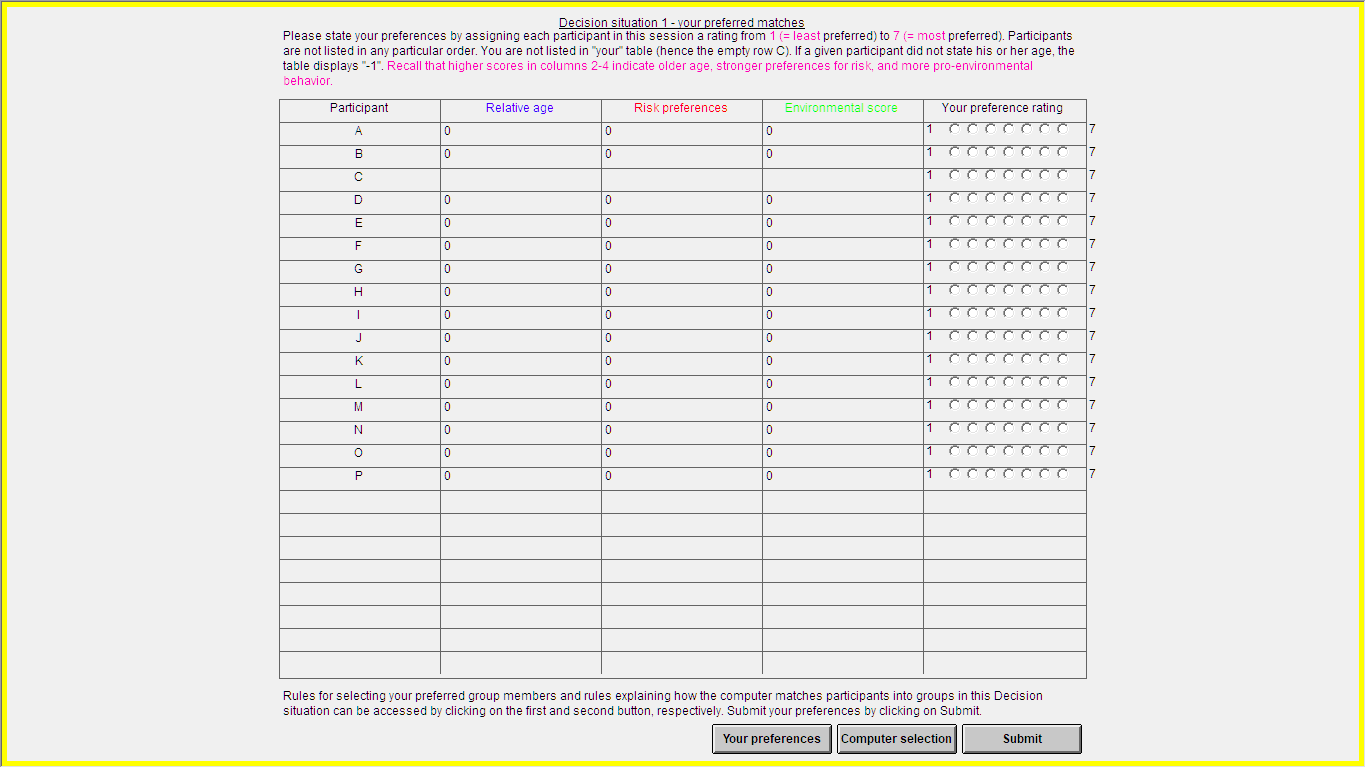

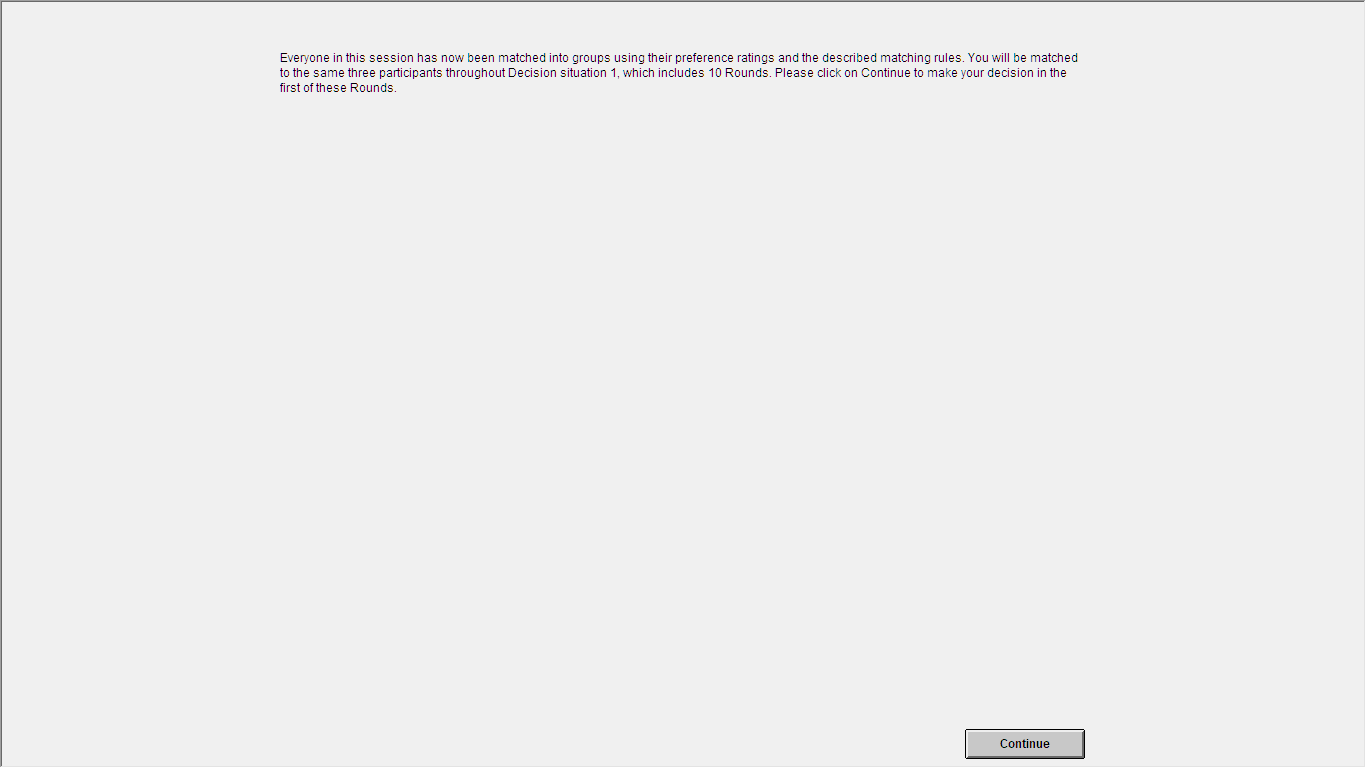

Supplement: Supplementary file 1 [file Data_Sheet_1.docx]
