# Supplementary material for: Is environmental behavior related to economic risk preferences? An exploratory case by case analysis
Source: Front Psychol. 2023 Aug 3;14:1212685. doi: 10.3389/fpsyg.2023.1212685 (PMC10434762; doi:10.3389/fpsyg.2023.1212685)
Supplement: Supplementary file 2 [file Data_Sheet_2.docx]

Online Appendix B: Coding of environmental behavior responses

Table B1 presents the complete list of items and the coding of all possible responses, incorporating coding decisions outlined under the table. Participants’ responses are scored so that higher scores indicate performing pro-environmental behavior.

Table B1: Environmental behavior items and response coding guide

| Environmental behavior (short label, used in Table 1 in the manuscript) | Item wording | Response option 1 and its coding | Response option 2 and its coding | Response option 3 and its coding |
| --- | --- | --- | --- | --- |
| Recycling paper | Do you collect and recycle used paper? | Yes = 1 | No = 0 |  |
| Composting | Do you use a compost bin? | Yes = 1 | No = 0 |  |
| Showering | Do you rather shower than take a bath? | Yes = 1 | No = 0 |  |
| Laundry | Do you wait to have a full load before doing your laundry? | Yes = 1 | No = 0 |  |
| Heater | Is the heater in your house shut off late at night? | Yes = 1 | No = 0 |  |
| Standby | Do you switch off or disconnect electric devices completely when not using them (not using standby)? | Yes = 1 | No = 0 |  |
| Room temperature | Do you lower the room temperature when you are not home for longer periods? | Yes = 1 | No = 0 |  |
| Electronic devices | When buying new electronic devices, do you make sure to buy the ones with the lowest energy use? | Yes = 1 | No = 0 |  |
| Refrigerator | Are you de-icing the refrigerator regularly? | Yes = 1 | No = 0 | I share the refridgerator with other people and someone else is responsible for this = coded as missing |
| Green electricity | Are you getting your electricity provider from a provider that guarantees 100% regenerative energy production? | Yes = 1 | No = 0 | I can not influence at all from which provider to get electricity = coded as missing |
| Dryer | Do you use a tumble dryer to dry clothes? | Yes = 0 | No = 1 | I do not have a tumble dryer = 1 |
| Detergent | Do you use phosphate-free laundry detergent? | Yes = 1 | No = 0 |  |
| Eating meat | Do you eat meat? | Yes = 0 | No = 1 |  |
| Local food | Do you prefer to buy local food products? | Yes = 1 | No = 0 |  |
| Shopping bags | Do you reuse your shopping bags? | Yes = 1 | No = 0 |  |
| Reusable cups | Do you bring your own coffee cup to school/work (instead of using cups from the coffee machine)? | Yes = 1 | No = 0 | I do not drink coffee at school/work = coded as missing |
| Reusable bottles | Do you usually buy beverages in reusable bottles? | Yes = 1 | No = 0 |  |
| Public transportation | Do you walk, ride a bicycle, or take public transportation to work or school? | Yes = 1 | No = 0 |  |
| Fuel efficiency | Do you drive a car with low fuel consumption or an alternative fuel car (e.g., an electric vehicle)? | Yes = 1 | No = 0 | I do not drive = 1 |
| Driving defensively | Do you drive defensively your car defensively to safe fuel? | Yes = 1 | No = 0 | I do not drive = 1 |
| Friends | Do you often talk with friends about problems related to the environment? | Yes = 1 | No = 0 |  |
| Environmental organization | Are you a member of an environmental organization? | Yes = 1 | No = 0 |  |
| Taxes | Do you support higher taxes for environmentally damaging behavior? | Yes = 1 | No = 0 |  |
| Incentives | Do you support financial incentives for environmentally beneficial technology? | Yes = 1 | No = 0 |  |
| Voting | Do you vote for parties that have environmental topics high on their agenda? | Yes = 1 | No = 0 |  |
| Batteries | Do you return dead batteries to collection points for dangerous waste? | Yes = 1 | No = 0 | I do not use batteries = coded as missing |
| Organic dairy | Do you buy organic dairy products? | Yes = 1 | No = 0 | I do not eat dairy products = coded as missing |
| Organic meat | Do you buy organic meat products? | Yes = 1 | No = 0 | I do not eat meat products = 1 |

Comments on coding:

For item “Are you de-icing the refrigerator regularly?”, the response “I share the refrigerator with other people and someone else is responsible for this” was coded as missing, as it did not unambiguously reflect a pro- or anti-environmental behavior. We acknowledge that this coding decision (and similar coding decisions below) reflect a judgment call on our part.

For item “Are you getting your electricity from a provider that guarantees 100% regenerative energy production?”, the response “I can not influence at all from which provider to get electricity” was coded as missing.

For item “Do you use a tumble dryer to dry clothes?”, the response “I do not have a tumble dryer” was recoded as “No”.

For item “Do you bring your own coffee (tea) cup to school/work (instead of using cups from the coffee machine)?”, the response “I do not drink coffee (tea) at school/work” was coded as missing.

For item “Do you drive a car with low fuel consumption or an alternative fuel car (e.g., an electric vehicle)?”, the response “I do not drive” was assigned the same value as “Yes” (i.e., both being coded as the maximally pro-environmental response).

For item “Do you drive your car defensively to safe fuel?”, the response “I do not drive” was assigned the same value as “Yes” (i.e., both being coded as the maximally pro-environmental response).

For item “Do you return dead batteries to collection points for dangerous waste?”, the response “I do not use batteries” was coded as missing.

For item “Do you buy organic dairy products?”, the response “I do not eat dairy products” was coded as missing.

For item “Do you buy organic meat products?”, the response “I do not eat meat products” was assigned the same value as “Yes” (i.e., both being coded as the maximally pro-environmental response).
